# Ruthenium indenylidene “1^st^ generation” olefin metathesis catalysts containing triisopropyl phosphite

**DOI:** 10.3762/bjoc.11.166

**Published:** 2015-09-01

**Authors:** Stefano Guidone, Fady Nahra, Alexandra M Z Slawin, Catherine S J Cazin

**Affiliations:** 1 EaStCHEM School of Chemistry, University of St Andrews, St Andrews, UK, KY16 9ST, UK

**Keywords:** 1^st^ generation, indenylidene, metathesis, phosphite, ruthenium

## Abstract

The reaction of triisopropyl phosphite with phosphine-based indenylidene pre-catalysts affords “1^st^ generation” *cis*-complexes. These have been used in olefin metathesis reactions. The *cis*-Ru species exhibit noticeable differences with the *trans*-Ru parent complexes in terms of structure, thermal stability and reactivity. Experimental data underline the importance of synergistic effects between phosphites and L-type ligands.

## Introduction

**Figure 1:**
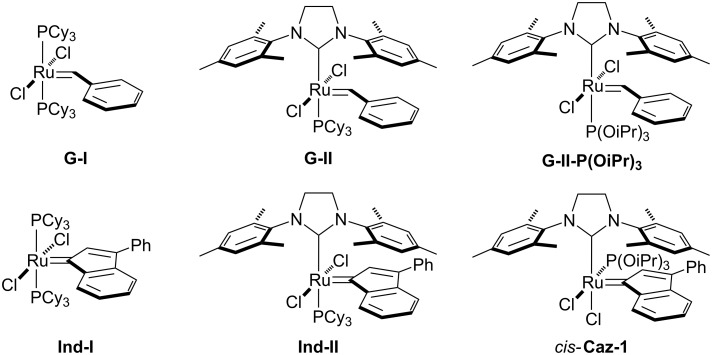
Examples of ruthenium complexes used in olefin metathesis reactions.

The olefin metathesis reaction is a powerful tool for C–C bond formation in the synthesis of highly valuable organic compounds [1–4]. Protocols involving W-, Mo- and Ru-based pre-catalysts can shorten or provide alternative synthetic pathways for the synthesis of natural products displaying complex chemical structures [5–9]. Ru-based pre-catalysts are known to be more air-, moisture- and functional-group tolerant compared to early transition metal complexes [10–13]. In general, the commonly used Ru(II)-based pre-catalysts have five ligands in the metal coordination sphere and adopt a distorted square pyramidal geometry (Figure 1).

The basic components of this structure include two L-type ligands mutually *trans* (e.g., phosphines and *N*-heterocyclic carbene) and two halides. The apex of the pyramid is occupied by an alkylidene moiety, such as a benzylidene or an indenylidene. Mixed NHC/phosphine complexes (**G-II** and **Ind-II**) known as “2^nd^ generation” pre-catalysts generally display higher catalytic activity than “1^st^ generation” complexes (**G-I** and **Ind-I**) containing two phosphines [14–23]. The most common phosphine, so called “throw-away ligand”, is tricyclohexyl phosphine [10–23]. In other words, such phosphorus donor ligands dissociate from the metal center to afford the 14e^−^ active species [10–13,24]. In order to reduce the cost of the Ru-based pre-catalyst, our group has investigated the use of phosphites as an economical alternative to phosphines. The reaction of triisopropyl phosphite with the pyridine-containing indenylidene complex [RuCl_2_(Ind)(SIMes)(py)] (SIMes = *N*,*N’*-bis[2,4,6-(trimethyl)phenyl]imidazolidin-2-ylidene) afforded a Ru pre-catalyst displaying an unusual *cis*-geometry [25]. *cis-***Caz-1**, which is more thermodynamically stable than its *trans*-isomer represents a breakthrough in catalyst-design for metathesis reactions of challenging hindered substrates (Figure 1) [25–34]. The latent behavior exhibited by *cis-***Caz-1** can be of interest in fields such as polymer chemistry, where its thermally-switchable properties can be used to inhibit polymerization during the storage of monomer-catalyst mixtures, and/or to initiate polymerization on demand through use of a stimulus [35–36]. The use of this catalyst in the ring-closing metathesis (RCM) reaction gave excellent conversions of challenging substrates, even at low catalyst loadings. The high activity and robustness of *cis-***Caz-1** is derived from synergistic effects between the σ-donor ligand NHC and the π-acidic triisopropyl phosphite [25,37]. Subsequently, the benzylidene analogue **G-II-P(OiPr)_3_** was also reported. The latter displayed a typical *trans*-configuration, seen in other Ru pre-catalysts, and gave a similar catalytic activity to that of the phosphine-containing parent **G-II** [26].

Because of the recent interest in “1^st^ generation” complexes [9,38–40], previous findings concerning “2^nd^ generation” complexes [25–33] and the desire to further reduce catalyst cost, the aim of this contribution is to replace the phosphine ligands in **Ind-I** with the less expensive triisopropyl phosphite and to study the structural and catalytic properties of these new species.

## Results and Discussion

### Synthesis of [RuCl_2_(Ind)(PCy_3_){P(OiPr)_3_}] (**1**)

**Scheme 1:**
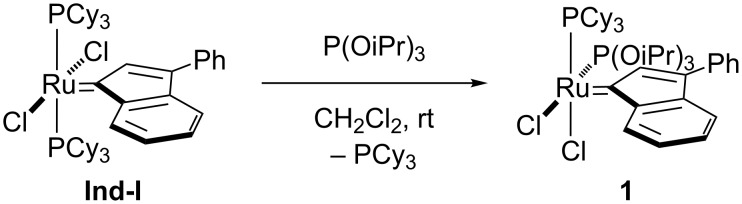
Synthesis of the mixed phosphine/phosphite complex **1**.

Attempts towards the synthesis of a mixed phosphine/phosphite complex involved the reaction of **Ind-I** with a stoichiometric amount of triisopropyl phosphite. Complex **1** was isolated in analytically pure form in 85% yield, after recrystallization, using a simple ligand exchange reaction (Scheme 1).

**Figure 2:**
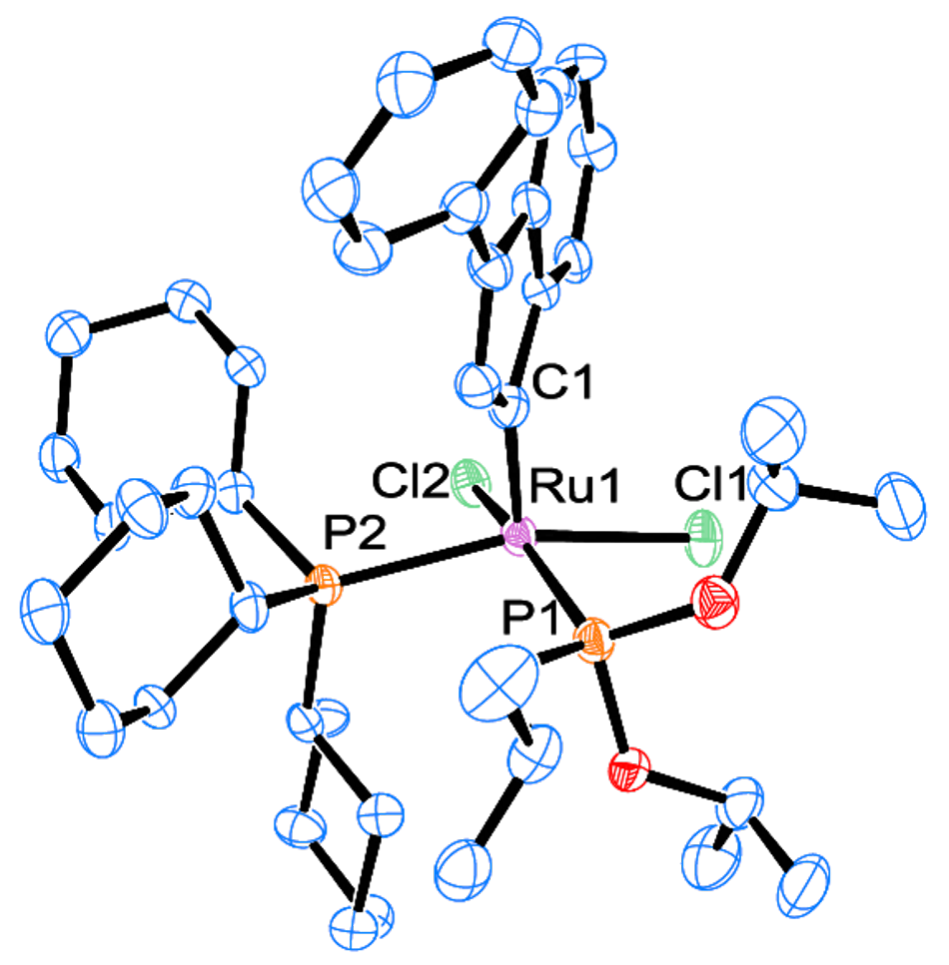
Molecular structure of mixed phosphine/phosphite complex **1**. Hydrogen atoms are omitted for clarity.

Similarly to the mixed NHC/phosphite species **Caz-1** [25], the *cis*-geometry is the most thermodynamically stable conformation for the phosphine/phosphite complex **1**. The corresponding *trans*-isomer was not isolated due to the fast isomerization occurring under the reaction conditions, although traces of transient species were detected by ^31^P-{^1^H} NMR spectroscopy (see Supporting Information File 1, section 4). The ^1^H NMR spectrum of **1** in CD_2_Cl_2_ showed the typical indenylidene proton system (characteristic doublet at low field, δ_H_ = 8.80 ppm). Coalescence of the aliphatic protons assigned to the cyclohexyl and the phosphite moieties was also observed at room temperature. These signals were resolved at a lower temperature (193 K). The ^13^C-{^1^H} NMR spectrum showed a doublet of doublets for the carbene carbon at δ_C_ = 290.3 ppm with two ^2^*J*_CP_ of 12.5 and 24.5 Hz (cf., *cis-***Caz-1**; 24.7 Hz) [25]. In the ^31^P-{^1^H} NMR spectrum, two doublets at 120.1 and 47.4 ppm with ^2^*J*_PP_ of 37.0 Hz were observed, consistent with a *cis*-disposition of the phosphorus donor ligands. This geometry was confirmed by X-ray diffraction analysis on a single crystal (Figure 2).

### Synthesis of [RuCl_2_(Ind){P(OiPr)_3_}_2_] (**2**)

**Scheme 2:**
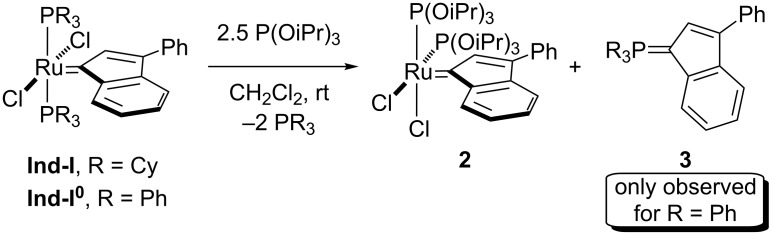
Synthesis of the bis-phosphite complex **2**.

The synthesis of the bis-phosphite species **2** was first attempted by the reaction of **Ind-I** with 2.5 equivalents of P(OiPr)_3_. Full conversion of the starting material was observed affording complex **2** (Scheme 2). Unfortunately, all attempts to purify **2** failed due to the presence of PCy_3_ decomposition products. The PPh_3_ adduct **Ind-I^0^** was then employed as alternative starting material for the ligand substitution reaction with the phosphite (Scheme 2) (see Supporting Information File 1, section 4). During the recrystallization from dichloromethane/pentane, compound **3** was detected as a decomposition product [41].

**Figure 3:**
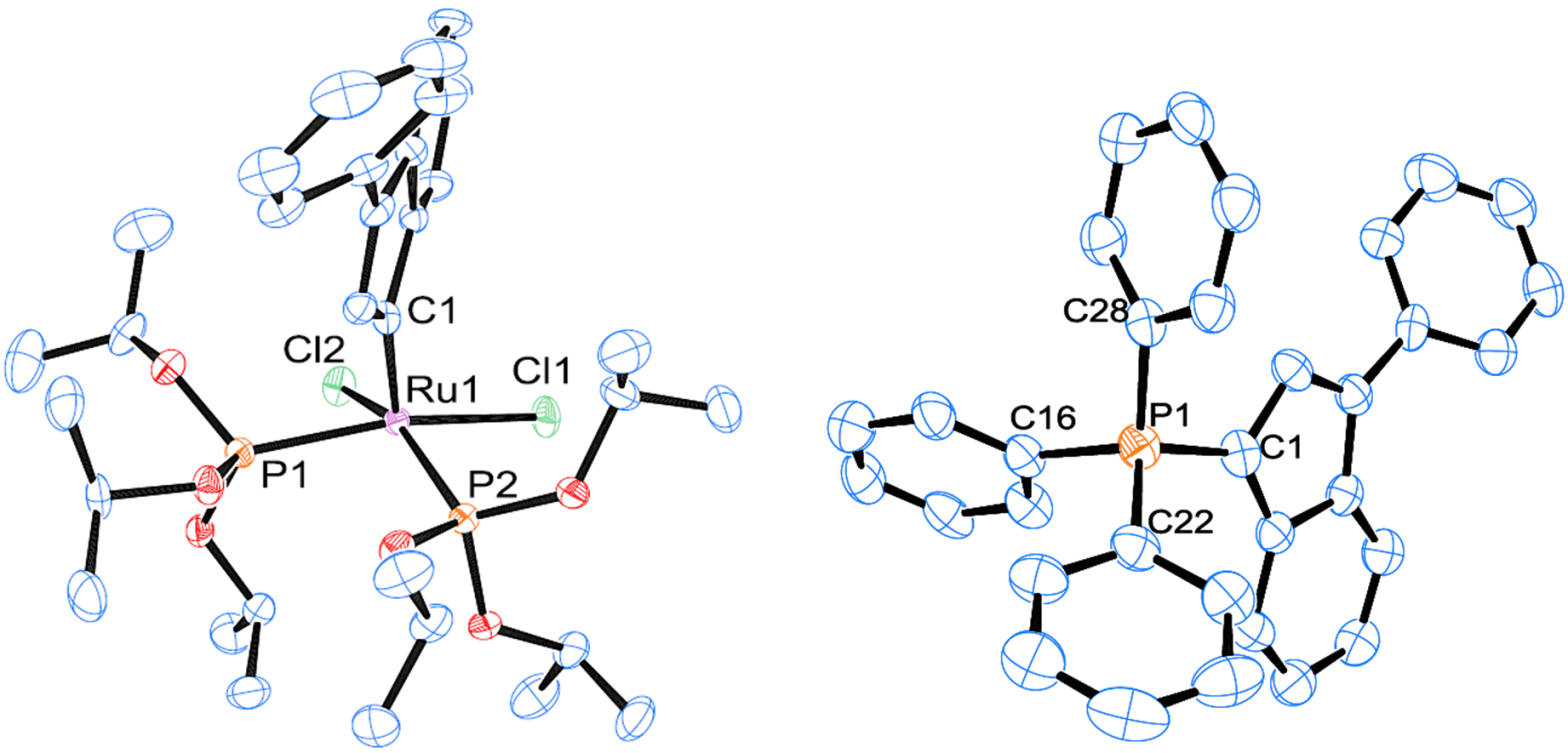
Molecular structure of **2** and the ylide **3**. Hydrogen atoms and solvent molecules are omitted for clarity. Selected bond distances (Å) and angles (°) (ESD) for compound **3**: P(1)–C(1), 1.713(5); P(1)–C(28), 1.795(5); P(1)–C(22), 1.805(5); P(1)–C(16), 1.811(5); C(1)–P(1)–C(28), 106.2(2); C(1)–P(1)-C(22), 113.1(3); C(28)–P(1)–C(22), 109.0(2); C(1)–P(1)–C(16), 113.3(2); C(28)–P(1)–C(16), 109.2(2); C(22)–P(1)–C(16), 105.9(2).

Due to the high solubility of this species and difficulties encountered in the purification process, product **2** was isolated with traces of compound **3** still present. In the ^1^H NMR spectrum of **2** in CD_2_Cl_2_ (with **3** present), the characteristic doublet at δ_H_ = 8.53 ppm for the indenylidene system was observed. The ^13^C-{^1^H} NMR spectrum contains a doublet of doublets for the carbene carbon at δ_C_ = 291.1 ppm with two similar ^2^*J*_CP_ of 22.0 Hz (cf. *cis-***Caz-1**, 24.7 Hz) [25]. In the ^31^P-{^1^H} NMR spectrum, two singlets at δ_P_ = 123.0 ppm and 10.9 ppm corresponding to **2** and **3**, respectively, were detected. Fortunately, we were able to cleanly isolate **3**, which allowed its full characterization and assignment. Crystals suitable for X-ray diffraction studies were grown for both species. These studies confirmed the relative *cis*-disposition of the phosphite ligands in **2** and the structure of **3** (Figure 3).

Complexes **1** and **2** display a rare distorted *cis*-square pyramidal geometry as observed in the case of *cis-***Caz-1** [25]. The *cis*-geometry differentiates these species from other “1^st^ generation” complexes that display the more common *trans*-geometry [14–17]. Comparing the details of the three structures in Table 1 (entries 1 to 3), the Ru–C_NHC_ bond distances are found shorter than Ru–P_phosphite_, and both of them are shorter than Ru–P_phosphine_ (Ru(1)–P(2) complex **1**) [34].

**Table 1:** Selected bond distances (Å) and angles (°) for **1**, **2** and *cis*-**Caz-1**.

Entry	Parameter	**1**	**2**	*cis*-**Caz-1**[25]

1	Ru(1)–C(1)	1.873(14)	1.869(3)	1.881(8)
2	Ru(1)–P(1)	2.239(4)	2.2300(9)	2.249(2)
3	Ru(1)–P(2)	2.387(4)	2.2663(8)	–
4	Ru(1)–C(NHC)	–	–	2.067(7)
5	Ru(1)–Cl(1)	2.398(4)	2.3999(9)	2.4036(18)
6	Ru(1)–Cl(2)	2.369(3)	2.3789(8)	2.3974(19)
7	P(1)–Ru(1)–P(2)	98.74(13)	97.34(3)	–
8	C(NHC)–Ru(1)–P(1)	–	–	100.06(19)
9	C(1)–Ru(1)–P(1)	90.2(5)	90.94(10)	90.5(2)
10	C(1)–Ru(1)–P(2)	94.3(4)	86.41(9)	–
11	C(1)–Ru(1)–C(NHC)	–	–	98.7(3)

From data listed in Table 1, the Ru–P bond appears stronger in the case of the Ru–phosphite than the Ru–phosphine scenario, suggesting the latter as the leaving ligand in catalysis (see Supporting Information File 1, section 5).

### Catalytic activity in ring-closing metathesis (RCM)

**Figure 4:**
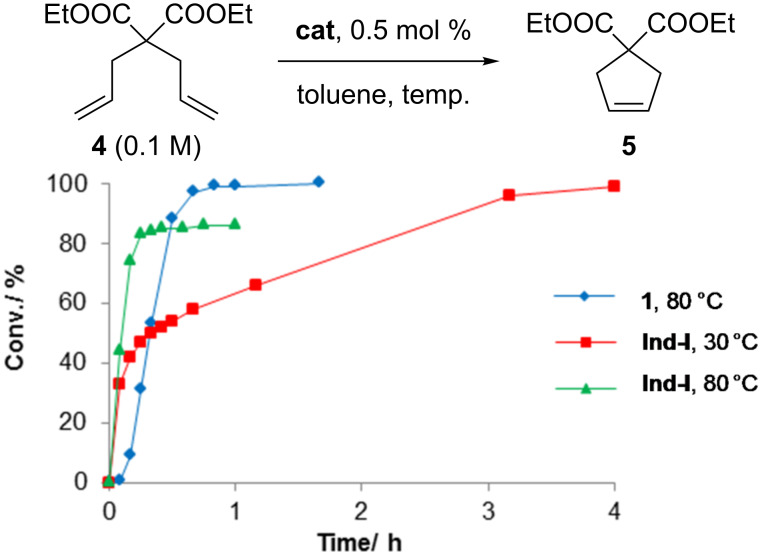
Reaction profiles of mixed phosphine/phosphite **1** and phosphine-based **Ind-I** in the RCM of **4** (lines are visual aids and not curve fits).

The reactivity of the mixed phosphine/phosphite complex **1** was first evaluated in the RCM of the easily cyclized diethyl diallylmalonate (**4**) (see Supporting Information File 1, section 2). The need for thermal activation for this pre-catalyst was clearly revealed by the low catalytic activity at 30–50 °C and the high conversion observed at 80 °C in toluene (0.1 mol % of **1**, 94% conv.). Contrary to **1**, the phosphine-based **Ind-I** initiates at 30 °C exhibiting good catalytic activity and undergoes fast decomposition at higher temperature with moderate conversion (see Supporting Information File 1, section 2). This trend was further studied by profiling reactions under catalytic conditions (Figure 4).

An induction period was observed for **1** at the early stage of the catalysis, a behavior similar to *cis-***Caz-1** [25], followed by a fast reaction with full conversion of the substrate at 80 °C in less than 50 min. These features prompted us to hypothesize an isomerization step from the *cis*-pre-catalyst **1** to the corresponding *trans* isomer as reported for *cis-***Caz-1** (see Supporting Information File 1, section 2) [25]. Under the same conditions, instant pre-catalyst initiation and fast decomposition of the active species were observed for the phosphine-based pre-catalyst **Ind-I** (86% conversion after 30 min). When the experiment was performed at 30 °C, **Ind-I** exhibited slower conversion of the substrate, reaching complete conversion after 4 h [22]. The reaction profiles show the importance of synergistic effects in the case of the mixed phosphine/phosphite system. Complex **1** is a thermally-switchable, latent pre-catalyst displaying higher thermal stability compared to the phosphine-based **Ind-I**.

Consequently, a brief study of the scope of the reaction was investigated employing “1^st^ generation” complexes **1** and **Ind-I** (Table 2).

**Table 2:** Scope of the reaction employing **1** and **Ind-I**.^a^

Entry	Substrate	Product	Pre-catalyst (mol %)	*T* (°C)	Conv. (%)^b^

1 2 3 4	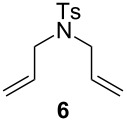	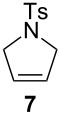	**1** (0.1) **1** (1) **Ind-I** (0.1) **Ind-I** (0.1)	80 80 80 30	<1 4 94 94
5 6 7	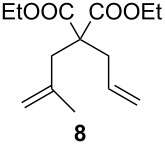	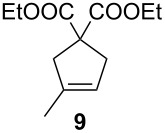	**1** (1) **Ind-I** (1) **Ind-I** (1)	80 80 30	79 (71) 33 53
8 9 10 11 12 13	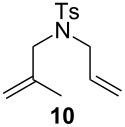	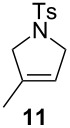	**1** (1) **1** (2) **Ind-I** (1) **Ind-I** (1) **Ind-I** (2) **Ind-I** (2)	80 80 80 30 80 30	41 41 22 21 30 77
14 15	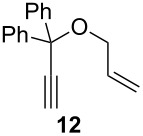	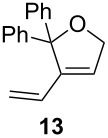	**1** (1) **Ind-I** (1)	80 30	78 98

^a^Reaction conditions: substrate (0.25 mmol), pre-catalyst (0.1 to 2 mol %), toluene (0.5 mL), 19 h. ^b^Conversions were determined by GC analysis. Isolated yields in parentheses.

The diene **6** was poorly converted by mixed PCy_3_/P(OR)_3_ complex **1**, whereas 94% conversion was obtained with **Ind-I** (Table 2, entries 1–4). In the case of tri-substituted diene **8**, a more challenging substrate compared to **6**, pre-catalyst **1** gave 79% conversion while **Ind-I** converted 33% of the substrate at 80 °C and 53% at 30 °C (Table 2, entries 5–7). The tosylamide derivative **10** was converted into product **11** by **1** (1 mol %) with 41% conversion (Table 2, entry 8). When the loading was increased to 2 mol %, no improvement in the conversion was detected (Table 2, entry 9). A higher catalytic activity was observed for **Ind-I** with 77% conversion when using 2 mol % pre-catalyst at 30 °C (Table 2, entry 13). Complex **1** was active in the ring-closing enyne metathesis (RCEYM) with 78% conversion of substrate **12** obtained with 1 mol % catalyst loading (Table 2, entry 14). A higher conversion of compound **12** was detected with **Ind-I** (98%, Table 2, entry 15).

## Conclusion

The influence of triisopropyl phosphite in Ru-based indenylidene “1^st^ generation” complexes has been presented. The mixed phosphine/phosphite complex **1** and the bis-phosphite complex **2** adopt distorted square pyramidal geometries with the P-donor ligands mutually *cis* as the most thermodynamically stable conformation. The isolation of the corresponding *trans*-isomers was not possible due to a fast isomerization process occurring during the synthesis of the complexes. Pre-catalyst **1** was found to be active in olefin metathesis reaction showing similarities with *cis*-**Caz-1** in terms of reactivity. Both pre-catalysts need thermal activation; they display an induction period in the reaction profiling and exhibit higher thermal stability compared to their phosphine-based analogues. In terms of catalytic efficiency, **Ind-I** was found more active than **1** unless higher thermal stability is needed. Indeed, in the case of the malonate derivative **8**, pre-catalyst **1** afforded the tri-substituted ring-closed product in 71% isolated yield. The similar structural and catalytic properties observed in the mixed phosphine/phosphite complex **1** and the mixed NHC/phosphite *cis***-Caz-1** suggest the importance of synergistic effects involving phosphites, an inexpensive alternative to phosphines for Ru-based pre-catalysts, and the L-type ligands, a concept that can be used to incite further improvements in catalyst design.

## Experimental

**Synthesis and characterization of [RuCl_2_(Ind)(PCy_3_){P(OiPr)_3_}]** (**1**)**:** Under an inert atmosphere of argon, triisopropyl phosphite (364 μL, 1.53 mmol) was added to a solution of **Ind-I** (1.414 g, 1.53 mmol) in dichloromethane (20 mL). The mixture was stirred for 24 h at room temperature and the solvent was then removed in vacuo. The crude product was recrystallized twice from dichloromethane/pentane. The solid was collected by filtration and washed with pentane (3 × 10, 2 × 15 mL). The product was obtained as a brownish red solid (1.116 g, 85%). During NMR experiments, peaks originating from the decomposition of the PCy_3_ were observed. ^1^H NMR of the mixture: (400 MHz, CD_2_Cl_2_) δ 1.08–1.33 (m, 6H, PCy_3_), 1.11 (d, ^3^*J*_HH_ = 6.3 Hz, 9H, CH-C*H*_3_), 1.30 (d, ^3^*J*_HH_ = 6.3 Hz, 9H, CH-C*H*_3_), 1.40–1.55 (m, 9H, PCy_3_), 1.60–1.85 (m, 15H, PCy_3_), 2.50 (m, 3H, C*H* PCy_3_), 4.55 (m, 3H, C*H*-CH_3_), 6.79 (s, 1H, H^2^), 7.27 (d, ^3^*J*_HH_ = 7.1 Hz, 1H, H^4^), 7.43 (dd, ^3^*J*_HH_ = 6.7 Hz, ^3^*J*_HH_ = 6.3 Hz, 1H, H^5^), 7.44 (dd, ^3^*J*_HH_ = 7.4 Hz, ^3^*J*_HH_ = 6.3 Hz, 2H, H^10^), 7.50 (dd, ^3^*J*_HH_ = 7.4 Hz, ^3^*J*_HH_ = 7.7 Hz, 1H, H^11^), 7.53 (dd, ^3^*J*_HH_ = 7.4 Hz, ^3^*J*_HH_ = 7.4 Hz, 1H, H^6^), 7.76 (d, ^3^*J*_HH_ = 7.3 Hz, 2H, H^9^), 8.80 (d, ^3^*J*_HH_ = 7.3 Hz, 1H, H^7^) ppm; ^13^C-{^1^H} NMR of the mixture (100.6 MHz, CD_2_Cl_2_) δ 24.3 (s, CH-*C*H_3_), 24.5 (d, ^3^*J*_CP_ = 4 Hz, CH-*C*H_3_), 26.9 (s, CH_2_ PCy_3_), 28.1 (d, ^2^*J*_CP_ = 11 Hz, CH_2_ PCy_3_), 28.4 (d, ^2^*J*_CP_ = 10 Hz, CH_2_ PCy_3_), 30.1 (s, CH_2_ PCy_3_), 30.6 (s, CH_2_ PCy_3_), 35.3 (s, CH PCy_3_), 71.4 (s, *C*H-CH_3_), 118.4 (s, C^4^), 127.0 (s, C^9^), 129.6 (s, C^10^), 129.7 (s, C^11^), 130.2 (s, C^7^), 130.3 (s, C^6^), 130. 7 (s, C^5^), 135.1 (s, C^8^), 136.5 (s, C^3^), 140.6 (s, C^3a^), 141.3 (dd, ^3^*J*_CP_ = 5.0 Hz, ^3^*J*_CP_ = 14.0 Hz, C^2^) 147.8 (s, C^7a^), 290.3 (dd, ^3^*J*_CP_ = 12.5 Hz, ^2^*J*_CP_ = 24.5 Hz, C^1^) ppm; ^31^P-{^1^H} NMR of the mixture (162 MHz, CD_2_Cl_2_) δ 120.1 (d, ^2^*J*_PP_ = 37.0 Hz, PCy_3_), 47.4 (d, ^2^*J*_PP_ = 37.0 Hz, P(O*^i^*Pr)_3_) ppm; anal. calcd for C_42_H_64_Cl_2_O_3_P_2_Ru: C, 59.29; H, 7.58; found: C, 59.45; H, 7.66. CCDC-889638 contains the supplementary crystallographic data for **1**. These data can be obtained free of charge from the Cambridge Crystallographic Data Centre via http://www.ccdc.cam.ac.uk/data_request/cif.

**Synthesis and characterization of [RuCl_2_(Ind){P(OiPr)_3_}_2_]** (**2**)**:** Under an inert atmosphere of argon, triisopropyl phosphite (65 μL, 0.28 mmol) was added to a solution of **Ind-I^0^** (0.100 g, 0.113 mmol) in dichloromethane (1.2 mL). The mixture was stirred for 24 h at room temperature and the solvent was removed in vacuo. The crude product was recrystallized from dichloromethane/pentane. The solid was collected by filtration and washed with pentane (3 × 3 mL). The product was obtained as a brownish green solid in a mixture with the phosphonium ylide **3** (0.032 g, 35%). ^1^H NMR of the mixture (400 MHz, CD_2_Cl_2_) δ 1.13 (d, ^3^*J*_HH_ = 6.3 Hz, 18H, CH-C*H*_3_), 1.31 (d, ^3^*J*_HH_ = 6.1 Hz, 18H, CH-C*H*_3_), 4.53 (m, ^3^*J*_HH_ = 6.3 Hz, 6H, C*H*-CH_3_), 7.18 (s, 1H, H^2^), 7.26 (d, ^3^*J*_HH_ = 6.9 Hz, 1H, H^4^), 7.40 (dd, ^3^*J*_HH_ = 7.1 Hz, ^3^*J*_HH_ = 7.1 Hz, 1H, H^5^), 7.43 (dd, ^3^*J*_HH_ = 7.4 Hz, ^3^*J*_HH_ = 7.4 Hz, 1H, H^6^), 7.46 (dd, ^3^*J*_HH_ = 7.4 Hz, ^3^*J*_HH_ = 7.4 Hz, 2H, H^10^), 7.53 (dd, ^3^*J*_HH_ = 7.4 Hz, ^3^*J*_HH_ = 7.4 Hz, 1H, H^11^), 7.77 (d, ^3^*J*_HH_ = 7.3 Hz, 2H, H^9^), 8.53 (d, ^3^*J*_HH_ = 7.0 Hz, 1H, H^7^) ppm; ^13^C-{^1^H} NMR of the mixture (100.6 MHz, CD_2_Cl_2_) δ 24.0 (s, CH-*C*H_3_), 24.4 (s, CH-*C*H_3_), 71.5 (m, *C*H-CH_3_), 119.0 (s, C^4^), 127.1 (s, C^9^), 129.6 (s, C^11^), 130.0 (s, C^10^), 130.2 (s, C^6^), 130.3 (s, C^7^), 131. 2 (s, C^5^), 134.8 (s, C^8^), 136.8 (s, C^3^), 140.3 (s, C^3a^), 140.7 (dd, ^3^*J*_CP_ = 8.5 Hz, C^2^) 150.2 (s, C^7a^), 291.1 (dd, ^2^*J*_CP_ = 22.0 Hz, ^2^*J*_CP_ = 22.0 Hz, C^1^) ppm; ^31^P-{^1^H} NMR (162 MHz, CD_2_Cl_2_) δ 123.0 (s, P(OiPr)_3_) ppm; CCDC-889639 contains the supplementary crystallographic data for **2**. These data can be obtained free of charge from the Cambridge Crystallographic Data Centre via http://www.ccdc.cam.ac.uk/data_request/cif.

**Characterization of Ph_3_P(Ind)** (**3**)**:** Under an inert atmosphere of argon, compound **3** was obtained as a side product from the reaction of **Ind-I^0^** (0.303 mg, 0.34 mmol, 1 equiv) with triisopropyl phosphite (179 μL, 0.75 mmol, 2.2 equiv) for 24 h. During recrystallization in a dichloromethane/pentane mixture, compound **3** was isolated as a yellow solid (63.4 mg, 41%). ^1^H NMR (400 MHz; CD_2_Cl_2_) δ 6.73 (d, ^2^*J*_HP_ = 4.9 Hz, 1H, H^2^), 6.77 (dd, ^3^*J*_HH_ = 7.5 Hz, ^3^*J*_HH_ = 7.5 Hz, 1H, H^6^), 6.89 (d, ^3^*J*_HH_ = 7.9 Hz, 1H, H^7^), 6.97 (dd, ^3^*J*_HH_ = 7.5 Hz, ^3^*J*_HH_ = 7.5 Hz, 1H, H^5^), 7.08 (dd, ^3^*J*_HH_ = 7.4 Hz, ^3^*J*_HH_ = 7.4 Hz, 1H, H^11^), 7.32 (dd, ^3^*J*_HH_ = 7.6 Hz, ^3^*J*_HH_ = 7.6 Hz, 2H, H^10^), 7.52–7.60 (m, 6H, C_6_H_5_), 7.61 (d, ^3^*J*_HH_ = 7.9 Hz, 2H, H^9^), 7.66–7.75 (m, 9H, C_6_H_5_), 7.96 (d, ^3^*J*_HH_ = 8.1 Hz, 1H, H^4^) ppm; ^13^C-{^1^H} NMR (100.6 MHz; CD_2_Cl_2_) δ 68.5 (d, ^1^*J*_CP_ = 121.6 Hz, C^1^), 118.2 (s, C^5^), 118.5 (s, C^6^), 118.7 (s, C^7^), 119.4 (s, C^4^), 121.2 (d, ^3^*J*_CP_ = 15.8 Hz, C^3^), 123.8 (s, C^11^), 125.6 (d, ^1^*J*_CP_ = 90.3 Hz, i-C_6_H_5_), 127.1 (s, C^9^), 127.8 (d, ^2^*J*_CP_ = 16.5 Hz, C^2^), 128.7 (s, C^10^), 129.5 (d, ^3^*J*_CP_ = 12.3 Hz, *m*-C_6_H_5_), 133.3 (d, ^4^*J*_CP_ = 2.7 Hz, *p*-C_6_H_5_), 134.2 (d, ^2^*J*_CP_ = 10.3 Hz, *o*-C_6_H_5_), 135.0 (d, ^3^*J*_CP_ = 13.9 Hz, C^3a^), 137.1 (d, ^2^*J*_CP_ = 13.2 Hz, C^7a^), 140.5 (s, C^8^) ppm; ^31^P-{^1^H} NMR (162 MHz; CD_2_Cl_2_) δ 10.9 (s, Ph_3_P(Ind)) ppm; HMRS (APCI): *m****/****z* calcd for [C_33_H_25_P + H] 453.18; found 453.1754. CCDC-889640 contains the supplementary crystallographic data for **3**. These data can be obtained free of charge from the Cambridge Crystallographic Data Centre via http://www.ccdc.cam.ac.uk/data_request/cif.

**General procedure for catalysis:** Substrates **6** [42], **8** [43], **10** [42], **12** [22], were synthesized following previous reports in the literature. Compound **4** was obtained from a commercial source and its purity confirmed prior to use. ^1^H NMR data of product **9** were compared to previously reported analyses [22].

A 5 mL screwcap-vial fitted with a septum equipped with a magnetic stirring bar was charged with the olefin (0.25 mmol) then purged with nitrogen, closed and introduced in a glovebox. The solvent and pre-catalyst (stock solution for <1 mol %, or weighed in the vial) were added to the reaction mixture (total amount of solvent 0.5 mL). Once out of the glovebox, the reaction mixture was heated at the desired temperature and stirred for 14 or 19 hours. The reaction mixture was then analyzed by GC and/or purified by flash chromatography.

**General procedure for kinetic experiments.** A Schlenk flask was charged with the olefin (0.5 mmol), then closed, placed under vacuum and introduced in a glovebox. The solvent was added (5 mL) and then the pre-catalyst was weighed and charged into the Schlenk flask. Out of the glovebox, the reaction was performed at the desired temperature. Samples were taken under nitrogen flow and quenched with ethyl vinyl ether. Data were obtained by GC analysis.

## Supporting Information

Crystallographic data for complexes **1**–**3** in CIF format can be obtained free of charge from the Cambridge Crystallographic Data Centre via http://www.ccdc.cam.ac.uk/data_request/cif (CCDC/889638-889640).

File 1 Crystallographic data for compounds **1**–**3**, NMR spectra of all the complexes, spectroscopic data.

File 2 CIF file for complex **2**.

File 3 CIF file for complex **3**.

File 4 CIF file for complex **4**.
