# Supplementary material for: Ruthenium indenylidene “1st generation” olefin metathesis catalysts containing triisopropyl phosphite
Source: Beilstein J Org Chem. 2015 Sep 1;11:1520–7. doi: 10.3762/bjoc.11.166 (PMC4578444; doi:10.3762/bjoc.11.166)
Supplement: File 1 — Crystallographic data for compounds 1–3, NMR spectra of all the complexes, spectroscopic data. [file Beilstein_J_Org_Chem-11-1520-s001.pdf]

Supporting Information

for

Ruthenium indenylidene “1st generation” olefin metathesis catalysis containing triisopropyl phosphite

Stefano Guidone, Fady Nahra, Alexandra M. Z. Slawin and Catherine S. J. Cazin*

Address: School of Chemistry University of St Andrews, St Andrews, UK, KY16 9ST
Fax: (+) 44 (0) 1334 463808
E-mail: cc111@st-andrews.ac.uk

*Corresponding author

Crystallographic data for compounds 1–3, NMR spectra of all the complexes, spectroscopic data.

Contents

1 General information S4
2 Optimization of catalytic conditions S5
3 NMR spectra S6
3.1 ^{1}H NMR (CD_2Cl_2) of **[RuCl$_2$(Ind)(PCy$_3$){P(OiPr)$_3$}]**, **1**, 293 K S6
3.2 ^{1}H NMR (CD_2Cl_2) of **1**, 193 K S6
3.3 ^{31}P-{^{1}H} NMR (CD_2Cl_2) of **1**, 293 K S7
3.4 ^{13}C-{^{1}H} NMR (CD_2Cl_2) of **1**, 293 K S7
3.5 ^{1}H NMR (CD_2Cl_2) of **[RuCl$_2$(Ind){P(OiPr)$_3$}$_2$] 2** in mixture with **3**, 293 K. S8
3.6 ^{31}P-{^{1}H} NMR (CD_2Cl_2) of **2** in mixture with **3**, 293 K S8
3.7 ^{13}C-{^{1}H} NMR (CD_2Cl_2) of **2** in mixture with **3**, 293 K S9
3.8 ^{1}H NMR (CD_2Cl_2) of Ph_3P(Ind), **3**, 293 K S9
3.9 ^{31}P-{^{1}H} NMR (CD_2Cl_2) of **3**, 293 K S10
3.10 ^{13}C-{^{1}H} NMR (CD_2Cl_2) of **3**, 293 K S10
3.11 ^{1}H NMR ($CDCl_3$) of **9**, 293 K S11
4 *trans*-Species S11
4.1 ^{31}P-{^{1}H} NMR (CD_2Cl_2) of **Ind-I^0** with 1 equiv P(OiPr)$_3$, 15 min, 293 K S11
4.2 ^{31}P-{^{1}H} NMR (CD_2Cl_2) of **Ind-I^0** with 1 equiv P(OiPr)$_3$, overnight, 293 K S12
4.3 ^{31}P-{^{1}H} NMR (CD_2Cl_2) of **Ind-I^0** with 2 equiv P(OiPr)$_3$, 15 min, 293 K S12
4.4 ^{31}P-{^{1}H} NMR (CD_2Cl_2) of **Ind-I^0** with 2 equiv P(OiPr)$_3$, overnight, 293 K S13
5 ^{31}P EXSY experiments with complex **1** S13
5.1 ^{31}P-{^{1}H} NMR (C_7D_8) of complex **1** with 5 equiv PCy$_3$, 128 scans, 293 K S14
5.2 ^{31}P EXSY (C_7D_8) of complex **1** with 5 equivequiv PCy$_3$, 128 scans, D8 = 0.6 s, 293 K . S14
5.3 ^{31}P EXSY (C_7D_8) of complex **1** with 5 equiv PCy$_3$, C_7D_8, 128 scans, D8 = 1.2 s, 293 K S15
5.4 ^{31}P-{^{1}H} NMR (C_7D_8) of complex **1** with 5 equiv PCy$_3$, 128 scans, 323 K S15
5.5 ^{31}P EXSY(C_7D_8) of complex **1** with 5 equiv. PCy$_3$, 128 scans, D8 = 0.6 s, 323 K S16
5.6 ^{31}P EXSY (C_7D_8) of complex **1** with 5 equiv PCy$_3$, 128 scans, D8 = 1.2 s, 323 K S16
5.7 ^{31}P-{^{1}H} NMR (C_7D_8) of complex **1** with 5 equiv PCy$_3$, 128 scans, 353 K S17
5.8 ^{31}P EXSY (C_7D_8) of complex **1** with 5 equiv PCy$_3$, 128 scans, D8 = 0.6 s, 353 K S17
5.9 ^{31}P EXSY (C_7D_8) of complex **1** with 5 equiv PCy$_3$, 128 scans, D8 = 1.2 s, 353 K S18
5.10 ^{31}P EXSY (C_7D_8) of complex **1** with 5 equiv PCy$_3$, 512 scans, D8 = 0.6 s, 353 K S18
5.11 ^{31}P EXSY (C_7D_8) of complex **1** with 5 equiv PCy$_3$, 512 scans, D8 = 1.2 s, 353 K S19
5.12 ^{31}P-{^{1}H} NMR (C_7D_8) of complex **1** with 5 equiv PCy$_3$, 128 scans, 293 K S19

6 HMRS analysis of product **3**........S20
7 Crystal data and structure refinement........S21
8 References........S21

1 General information

All reactions were performed under an inert atmosphere of argon or nitrogen using standard Schlenk line and glovebox techniques. Solvents were dispensed from a solvent purification system from Innovative Technology. ^{1}H, ^{13}C-{^{1}H} and ^{31}P-{^{1}H} NMR spectra were recorded on a Bruker AC300 or on a Bruker Avance 400 Ultrashield spectrometer at 298 K unless otherwise stated. Exchange spectroscopy experiments (EXSY) were carried out using a Bruker AVANCE 500 NMR spectrometer equipped with a QNP probe tuned for ^{31}P observation and ^{1}H decoupling. The temperature was controlled by a Bruker BVT unit. The 1D selective ^{31}P EXSY spectra[1] were acquired with a Bruker pulse program *selno* which was adjusted by applying ^{1}H *waltz16* decoupling during both acquisition and the selective ^{31}P excitation pulse. A standard 90° Gaussian pulse with a duration of 10 ms was used for selective excitation. The mixing time t_m (D8) ranged between 0.6 and 1.2 s. Gas chromatography analyzes were carried out using an Agilent 7890A system with a flame ionization detector and a 5%-phenylmethylpolysiloxane column (30 m, 320 μm, film: 0.25 μm). Flash chromatography was carried out using 40–63 μm silica. Elemental analyses were performed by the Elemental Analysis Service of the University of St Andrews or the Science Centre of the London Metropolitan University. HMRS analysis was performed by the EPSRC National Mass Spectrometry Service Centre at Swansea University. All reagents and commercial catalysts were purchased and used as received unless otherwise noted.

2 Optimization of catalytic conditions

Table S1: Optimization and pre-catalyst comparison in the RCM of diethyl diallylmalonate **4**.[a]

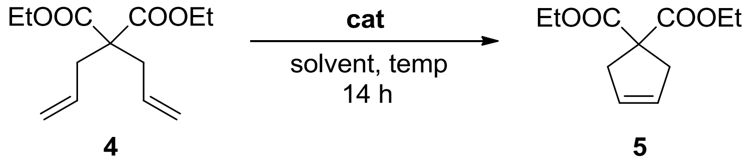

Entry	Solvent	Pre-catalyst (mol%)	*T* (°C)	Conv. (%)[b]
1	CH_2Cl_2	**1** (1)	30	4
2	CH_2Cl_2	**1** (1)	50	10
3	toluene	**1** (1)	30	2
4	toluene	**1** (1)	60	98
5	toluene	**1** (1)	80	> 99
6	toluene	**1** (1)	100	> 99
7	toluene	**1** (1)	120	95
8	toluene	**1** (0.1)	70	84
9	toluene	**1** (0.1)	80	94
10	toluene	**1** (0.1)	100	90
11	toluene	**1** (0.1)	120	82
12	toluene	**Ind-I** (0.1)	80	66
13	toluene	**Ind-I** (0.1)	30	98
14	toluene	**Ind-I^{0}** (0.1)	30	< 1
15	toluene	**Ind-I^{0}** (0.1)	80	97

[a]Reaction conditions: substrate (0.25 mmol), pre-catalyst (0.1 to 1 mol %), solvent (0.5 mL), 14 h.
[b]Conversions were determined by GC analysis, average of two runs.

3 NMR spectra

3.1 ^{1}H NMR (CD_2Cl_2) of [$RuCl_2$(Ind)(PCy_3){P(OiPr)$_3$}], 1, 293 K

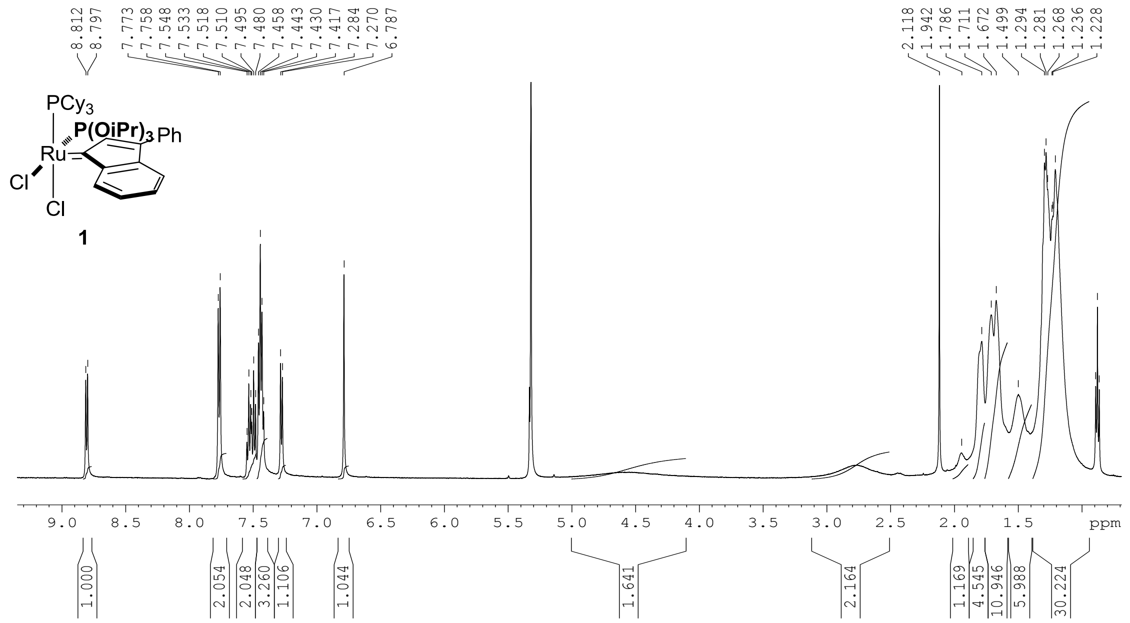

3.2 ^{1}H NMR (CD_2Cl_2) of 1, 193 K

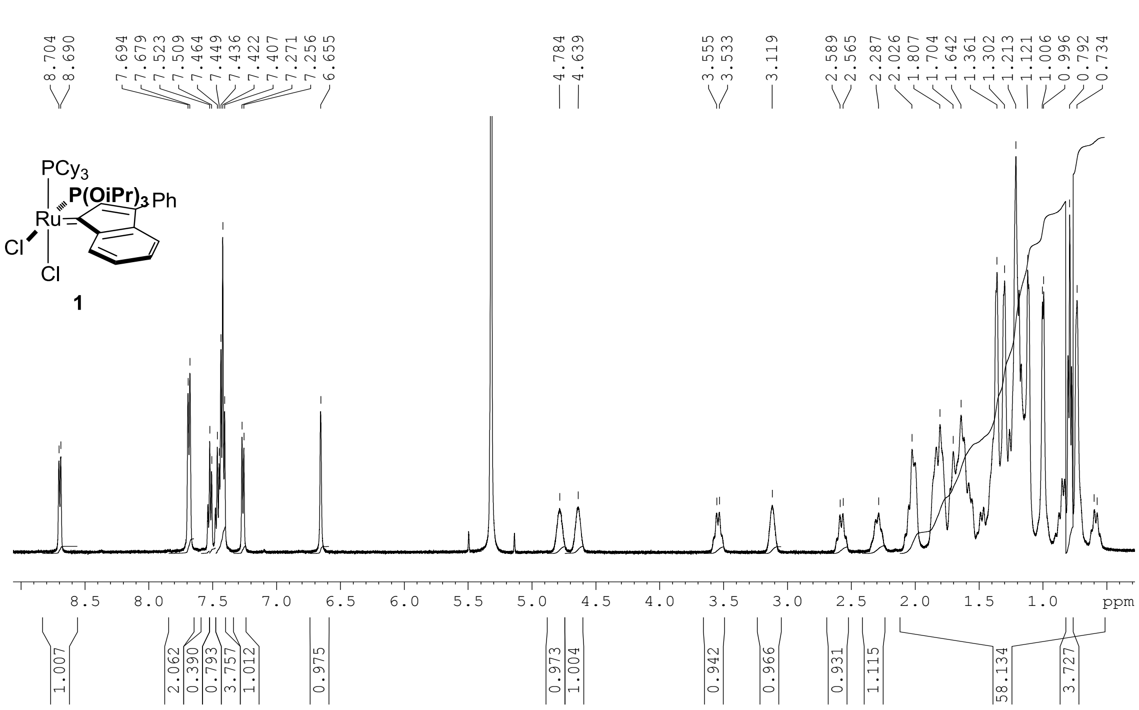

3.3 ^{31}P-{^{1}H} NMR (CD_2Cl_2) of 1, 293 K

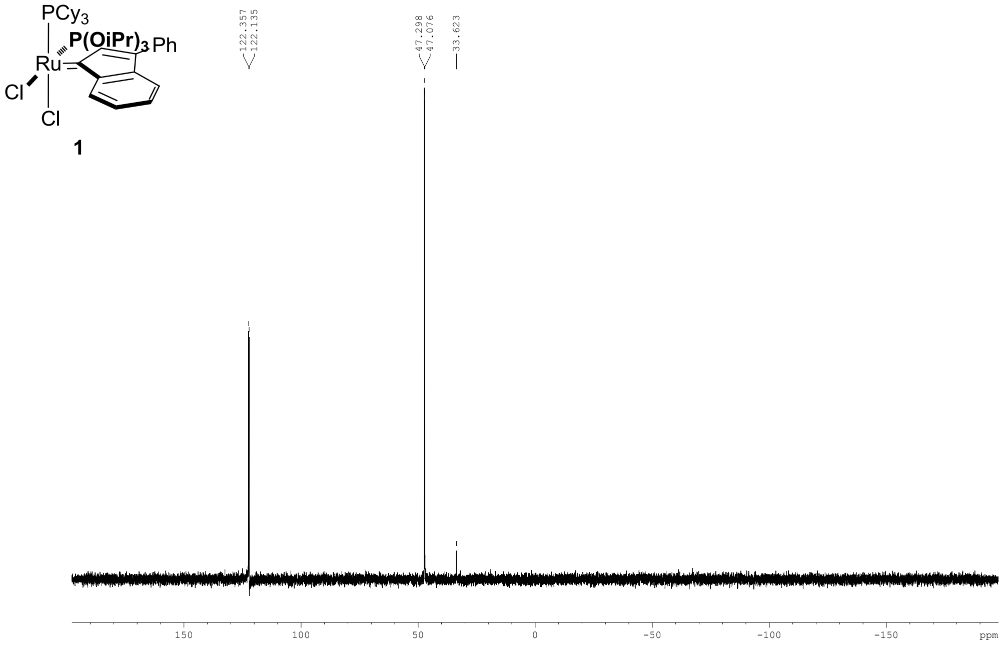

The phosphorous nuclei exhibit different relaxation times. Traces of impurities at 33.5 ppm with no influence on the elemental analysis.

3.4 ^{13}C-{^{1}H} NMR (CD_2Cl_2) of 1, 293 K

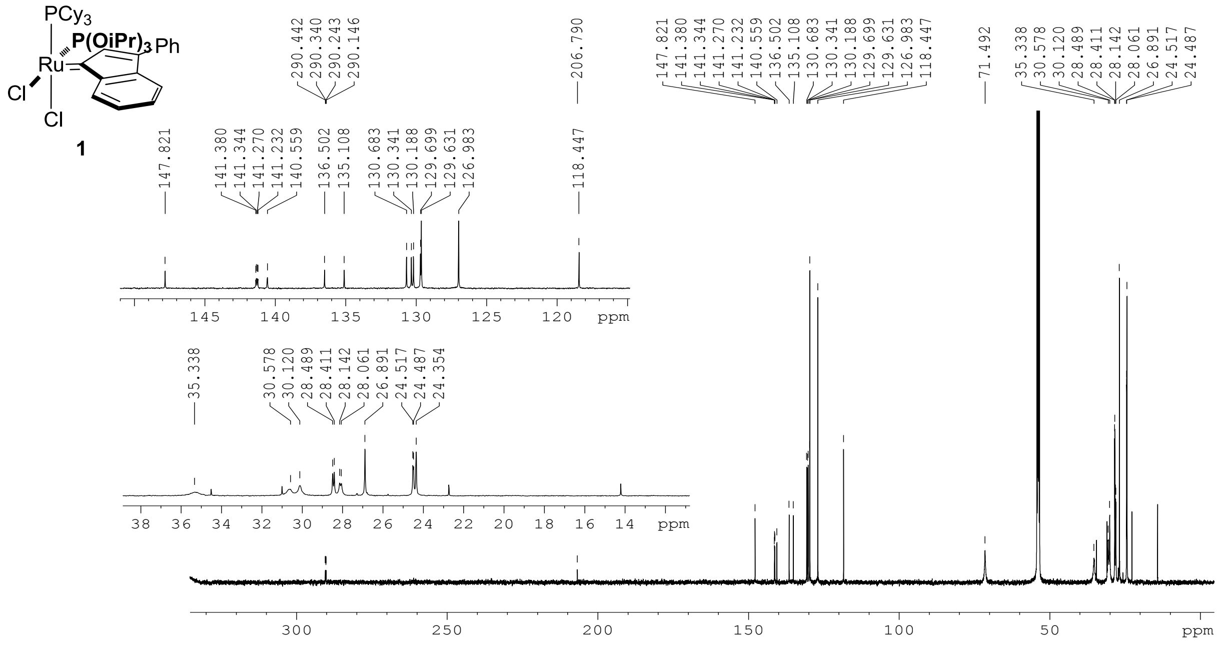

3.5 ^{1}H NMR (CD_2Cl_2) of $[RuCl_2(Ind)\{P(OiPr)_3\}_2]$ 2 in mixture with 3, 293 K.

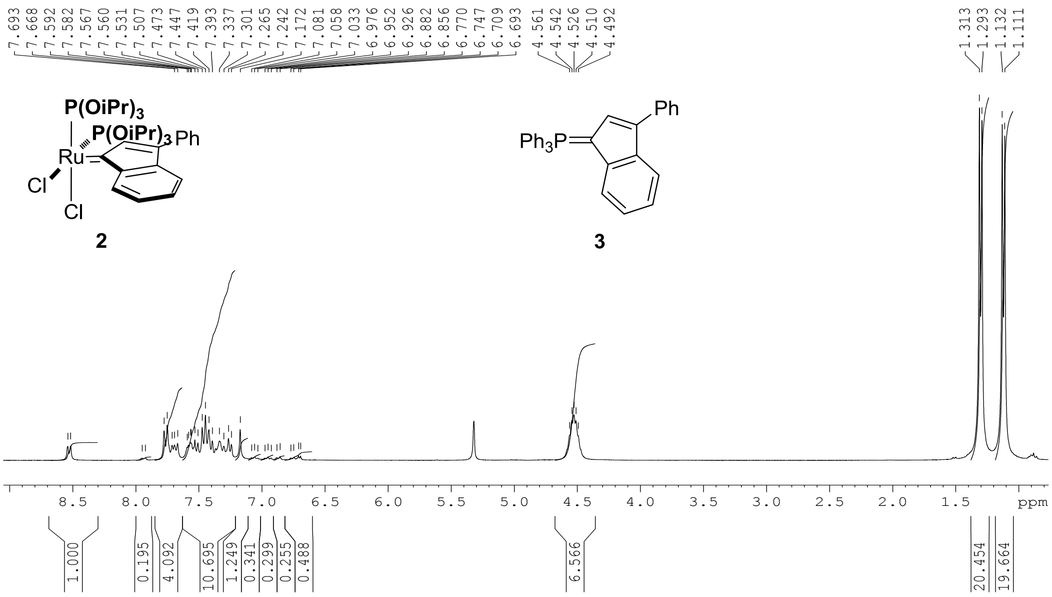

3.6 ^{31}P-{^{1}H} NMR (CD_2Cl_2) of 2 in mixture with 3, 293 K

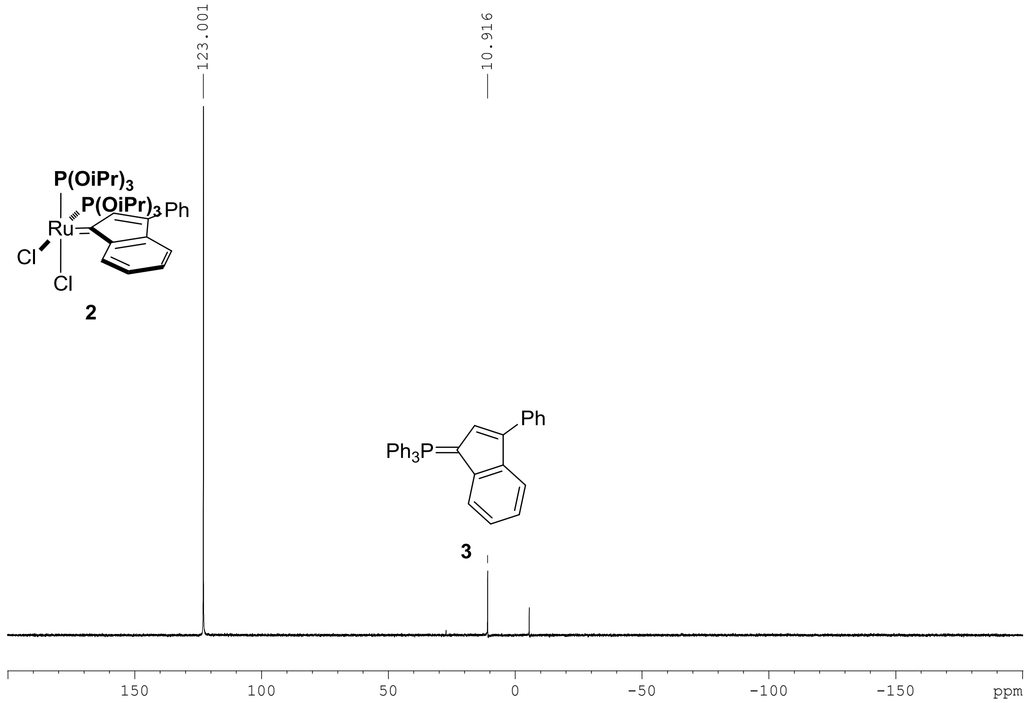

Traces of impurities detected at -3 ppm.

3.7 ^{13}C-{^{1}H} NMR (CD_2Cl_2) of 2 in mixture with 3, 293 K

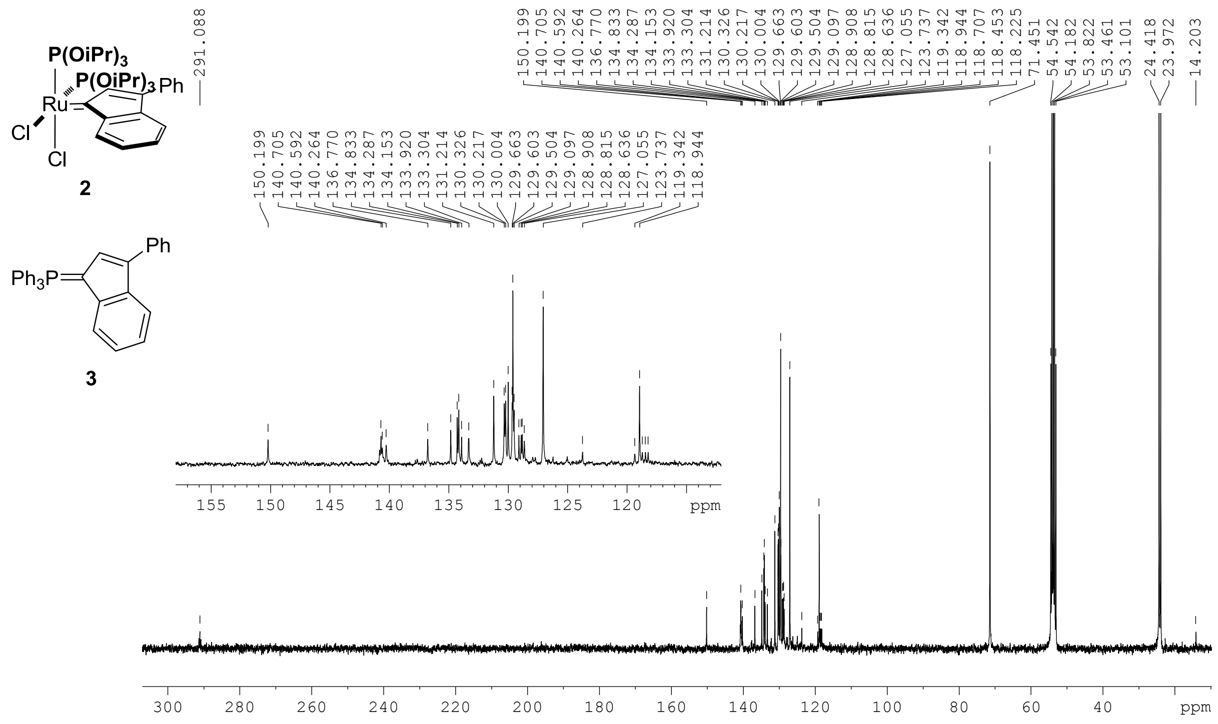

3.8 ^{1}H NMR (CD_2Cl_2) of Ph_3P(Ind), 3, 293 K

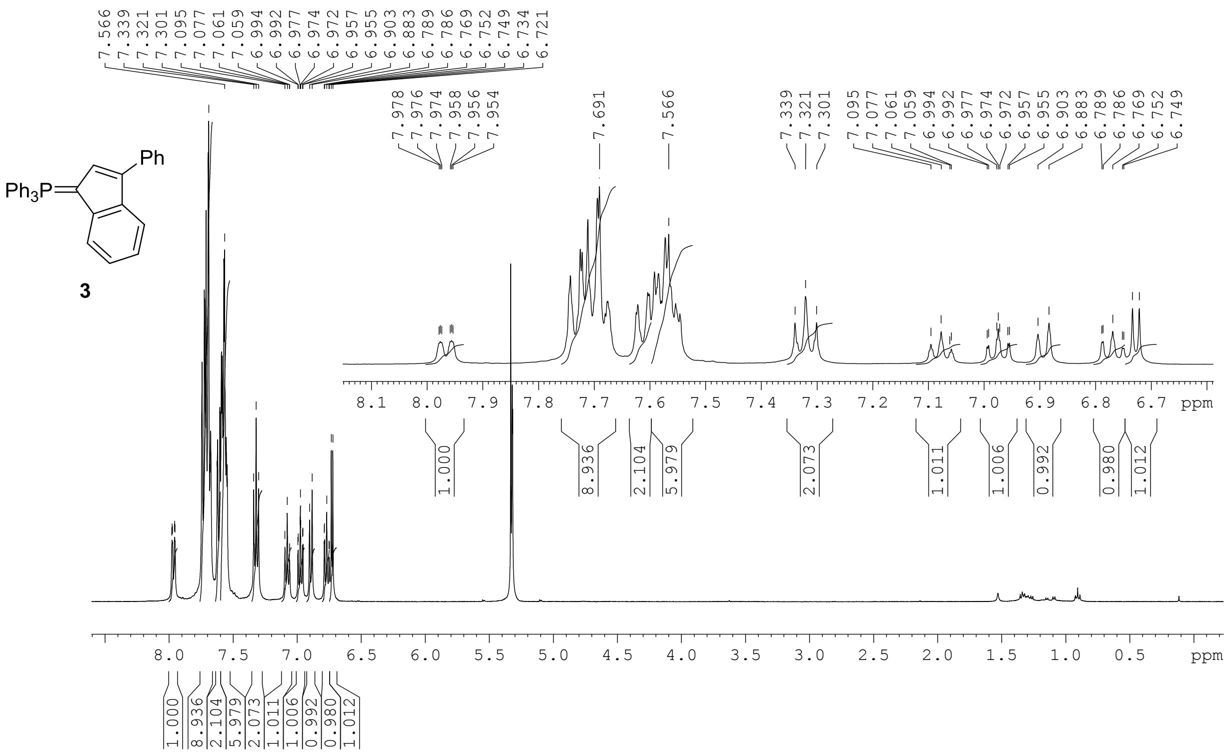

3.9 ^{31}P-{^{1}H} NMR (CD_2Cl_2) of 3, 293 K

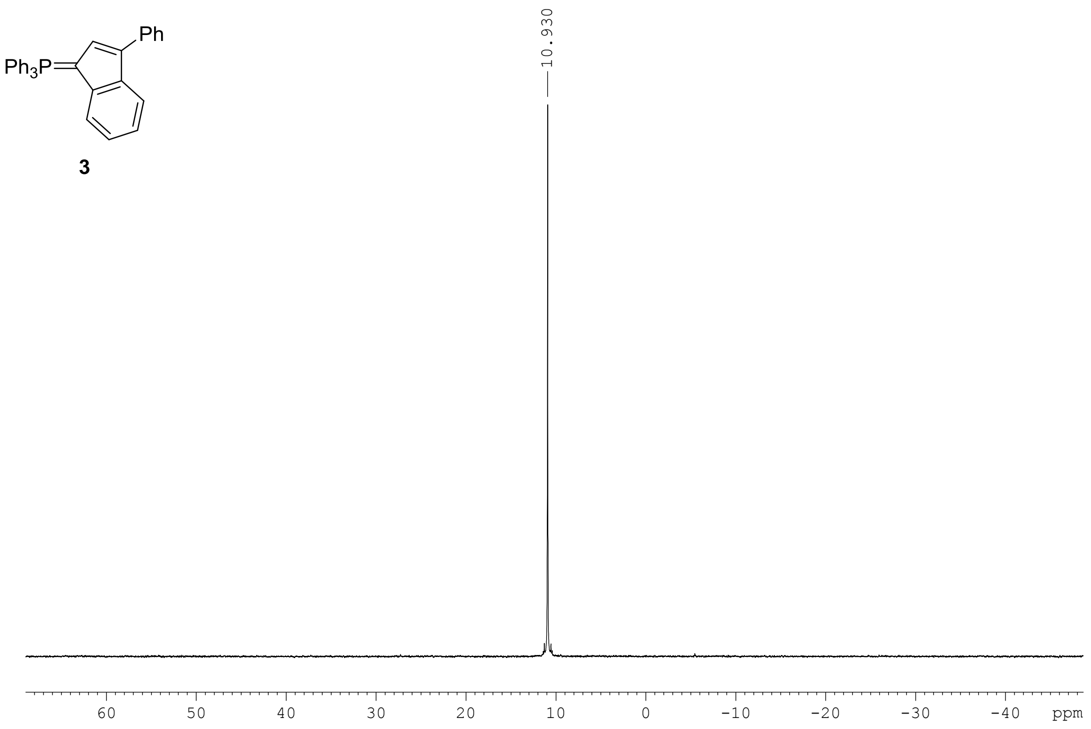

3.10 ^{13}C-{^{1}H} NMR (CD_2Cl_2) of 3, 293 K

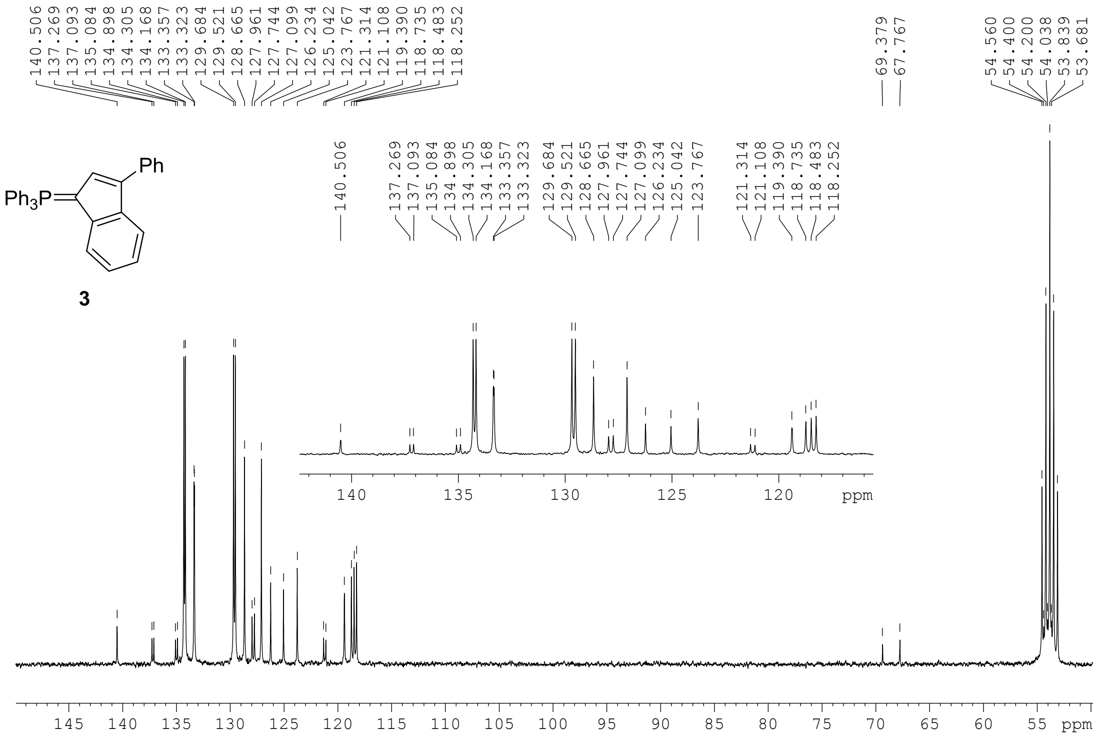

3.11 ^{1}H NMR ($CDCl_3$) of 9, 293 K

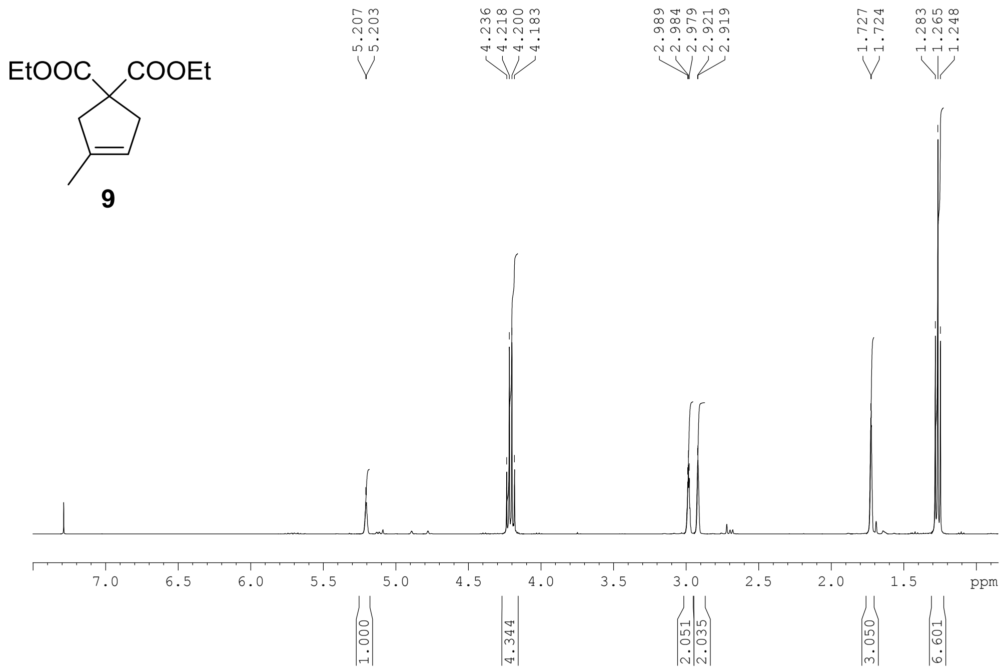

4 *trans*-Species

In a glovebox, an NMR tube was charged with **Ind-I^{0}** (20 mg, 0.023 mmol) and triisopropyl phosphite (5 µL, 0.026 mmol; 10 µL, 0.048 mmol) and dichloromethane (0.5 mL). The reaction was monitored by ^{31}P-{^{1}H} NMR spectroscopy.

4.1 ^{31}P-{^{1}H} NMR (CD_2Cl_2) of Ind-I^{0} with 1 equiv $P(OiPr)_3$, 15 min, 293 K

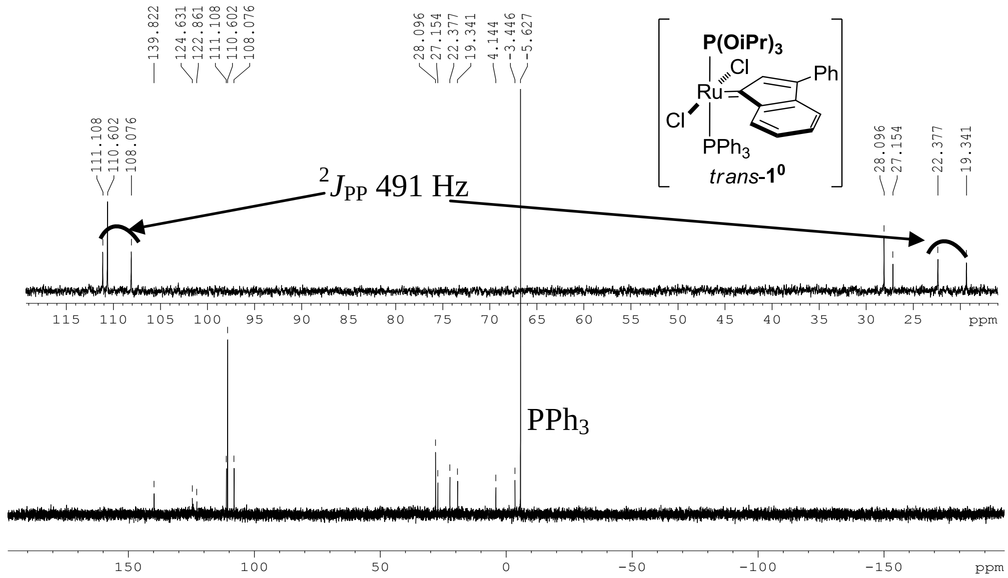

4.2 $^{31}P-\{^1H\}$ NMR (CD_2Cl_2) of Ind-I^0 with 1 equiv $P(OiPr)_3$, overnight, 293 K

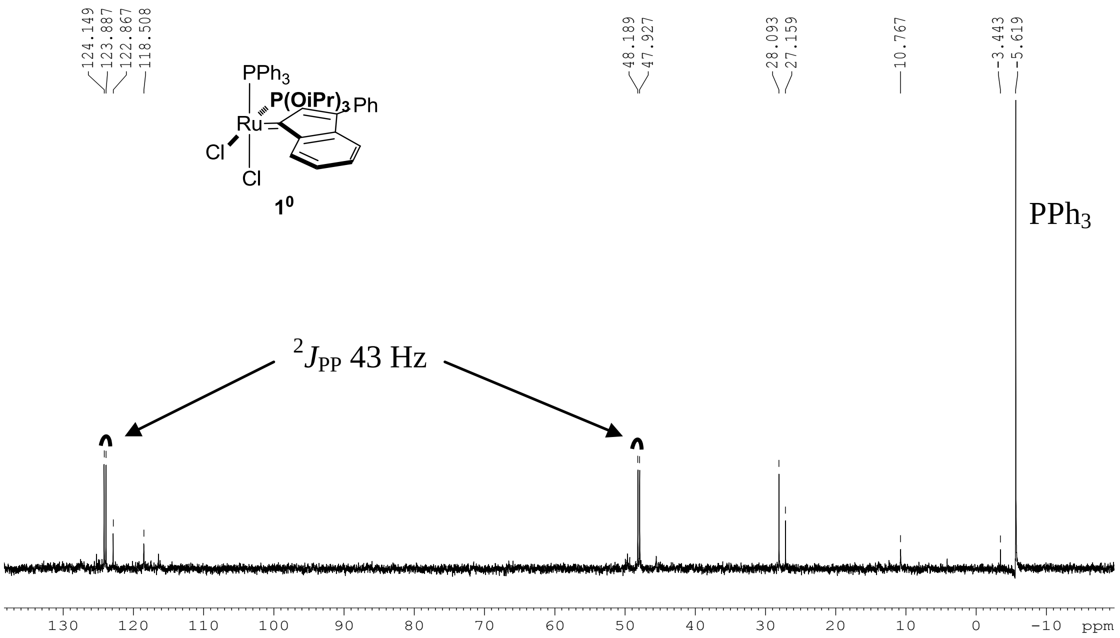

4.3 $^{31}P-\{^1H\}$ NMR (CD_2Cl_2) of Ind-I^0 with 2 equiv $P(OiPr)_3$, 15 min, 293 K

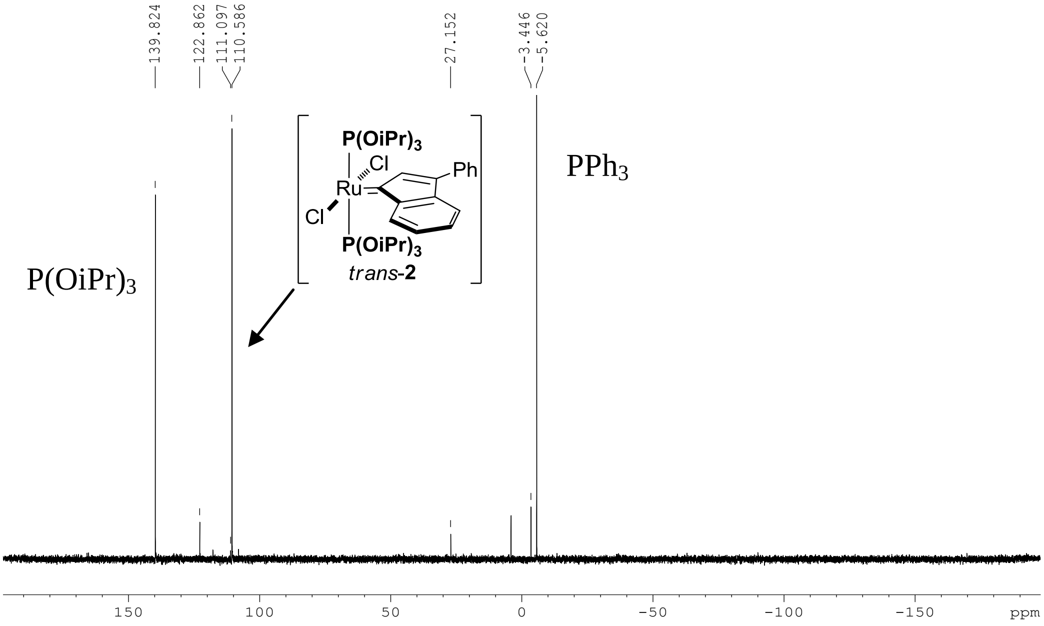

4.4 ^{31}P-{^{1}H} NMR (CD_2Cl_2) of Ind-I^0 with 2 equiv $P(OiPr)_3$, overnight, 293 K

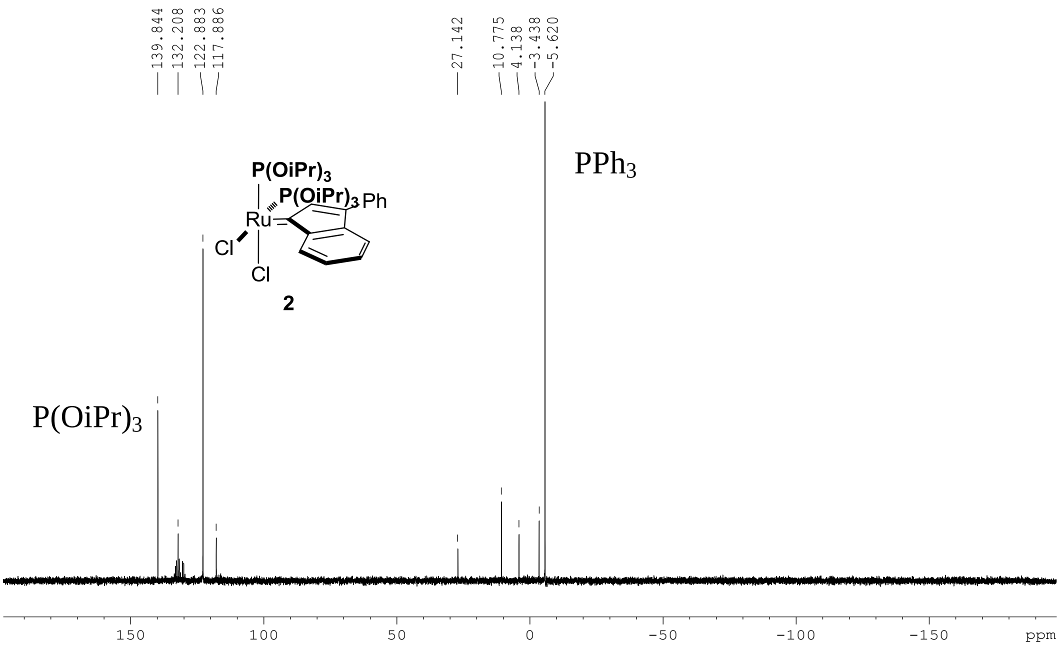

5 ^{31}P EXSY experiments with complex 1

General Procedure. In a glovebox, a NMR tube was charged with complex **1** (5.2 mg, 0.006 mmol, 1 equiv), PCy_3 (8.3 mg, 0.03 mmol, 5 equiv) and dissolved in toluene-d_8 (0.5 mL). ^{31}P-{^{1}H} NMR spectra were recorded before and after any ^{31}P EXSY experiment in order to monitor the composition of the solution. ^{31}P EXSY experiments were run at 293 K, 323 K, 353 K and back to 293 K with the same sample in sequence (see 5.1-5.12).

5.1 ^{31}P-{^{1}H} NMR (C_7D_8) of complex 1 with 5 equiv PCy_3, 128 scans, 293 K

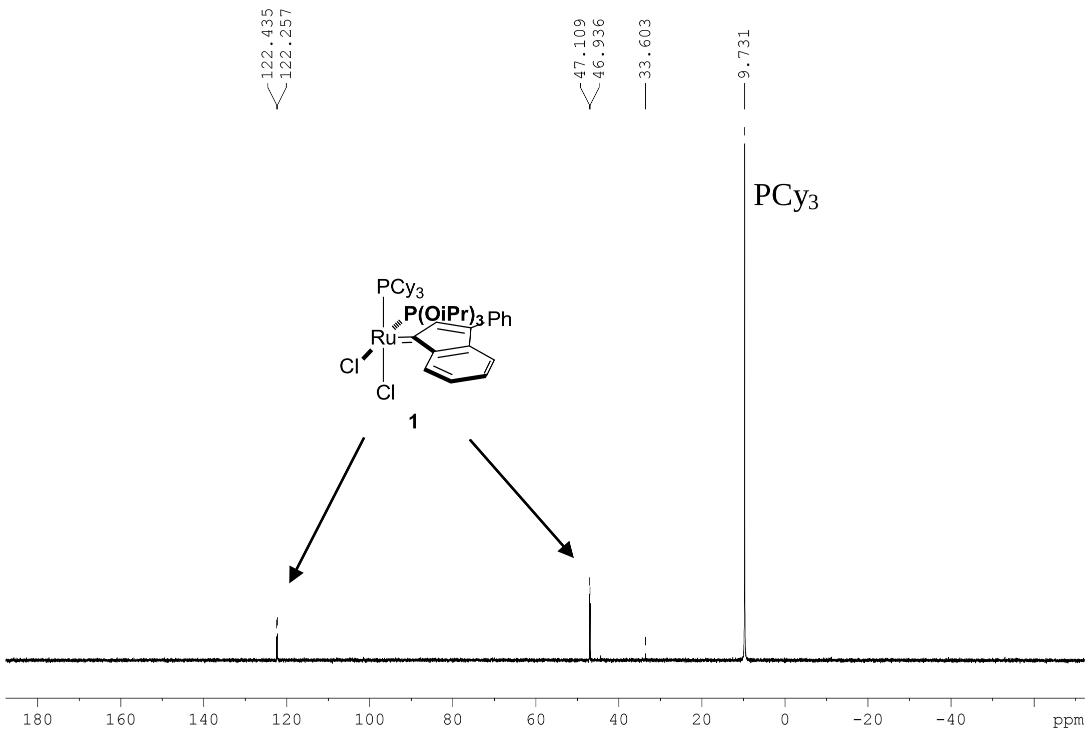

5.2 ^{31}P EXSY (C_7D_8) of complex 1 with 5 equiv PCy_3, 128 scans, D8 = 0.6 s, 293 K

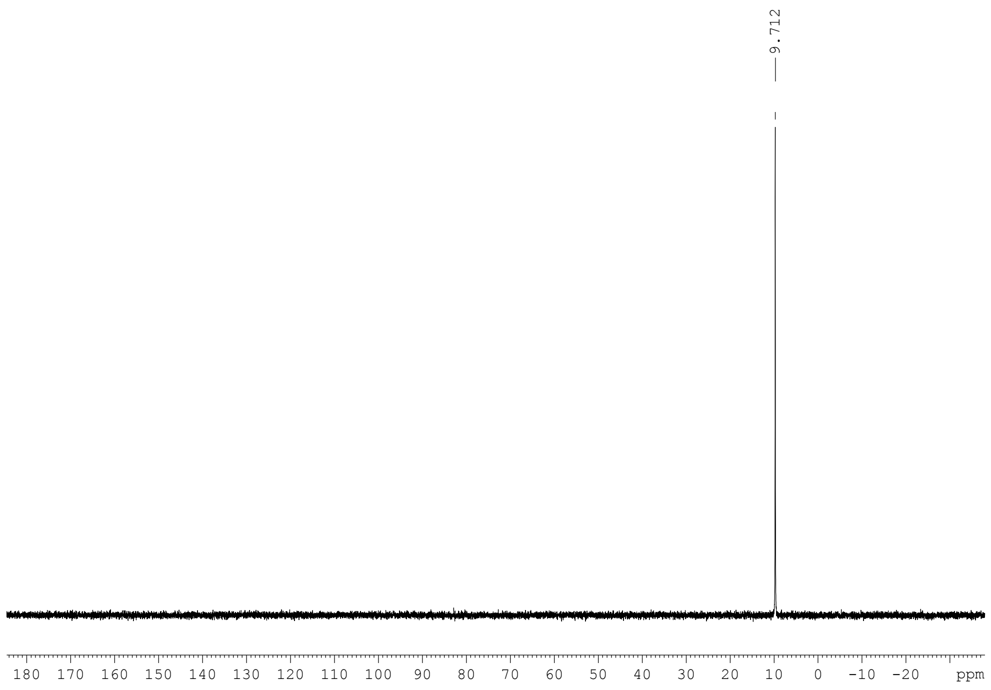

No exchange observed with complex **1**.

5.3 ^{31}P EXSY (C_7D_8) of complex 1 with 5 equiv PCy_3, C_7D_8, 128 scans, D8 = 1.2 s, 293 K

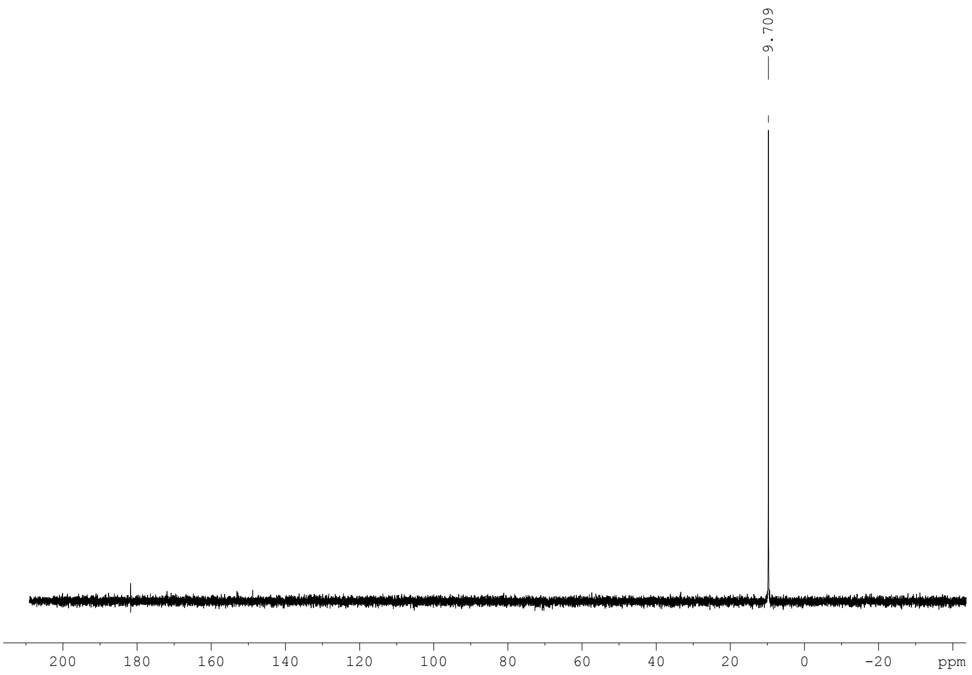

No exchange observed with complex **1**.

5.4 ^{31}P-{^{1}H} NMR (C_7D_8) of complex 1 with 5 equiv PCy_3, 128 scans, 323 K

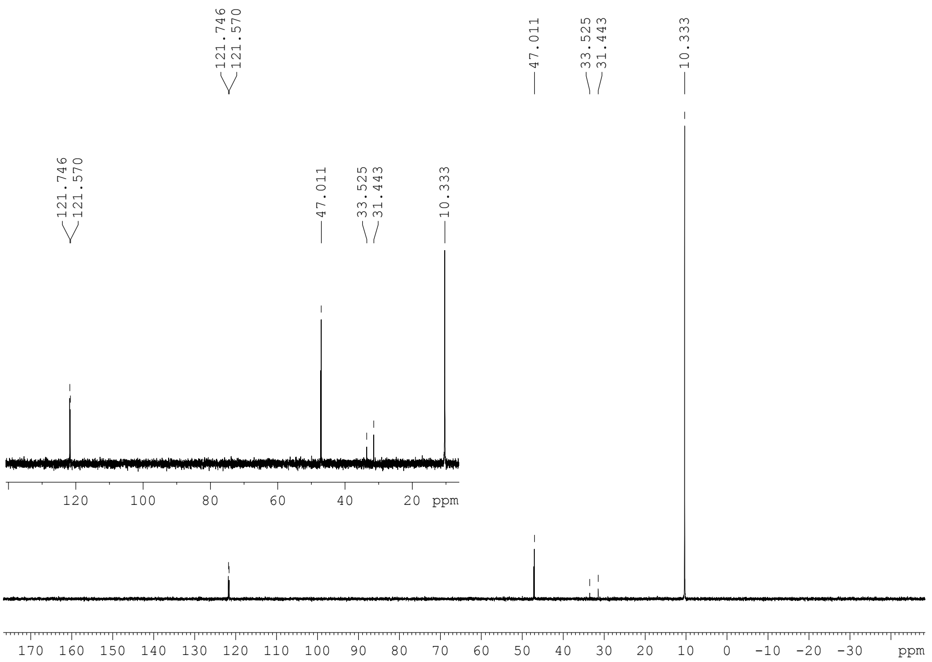

Reference spectrum of the NMR sample at 323 K. Some decomposition observed at 33.5 ppm.

5.5 ^{31}P EXSY(C_7D_8) of complex 1 with 5 equiv PCy_3, 128 scans, D8 = 0.6 s, 323 K

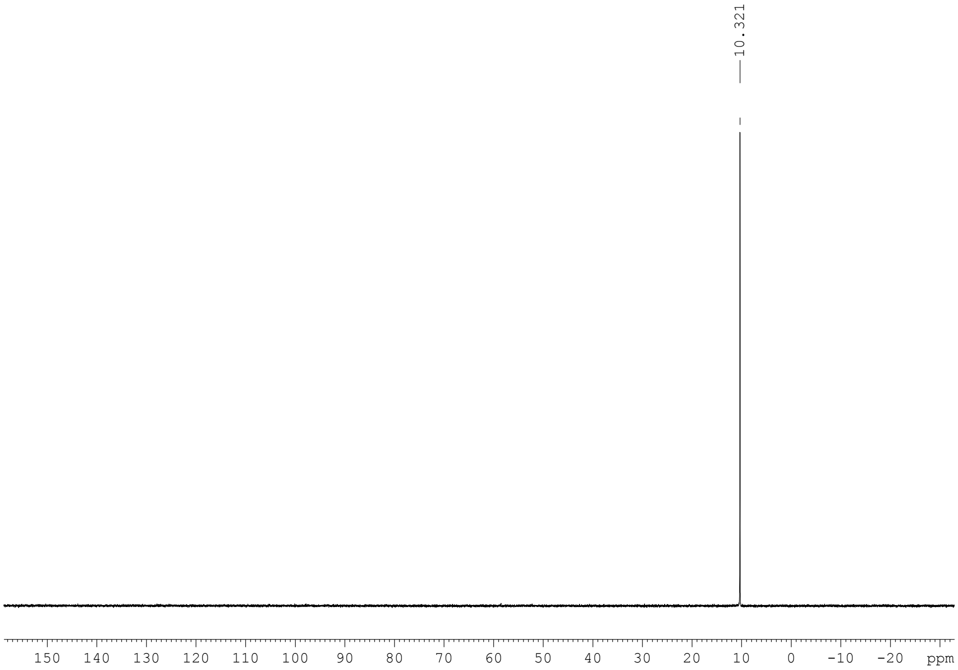

No exchange observed with complex **1**.

5.6 ^{31}P EXSY (C_7D_8) of complex 1 with 5 equiv PCy_3, 128 scans, D8 = 1.2 s, 323 K

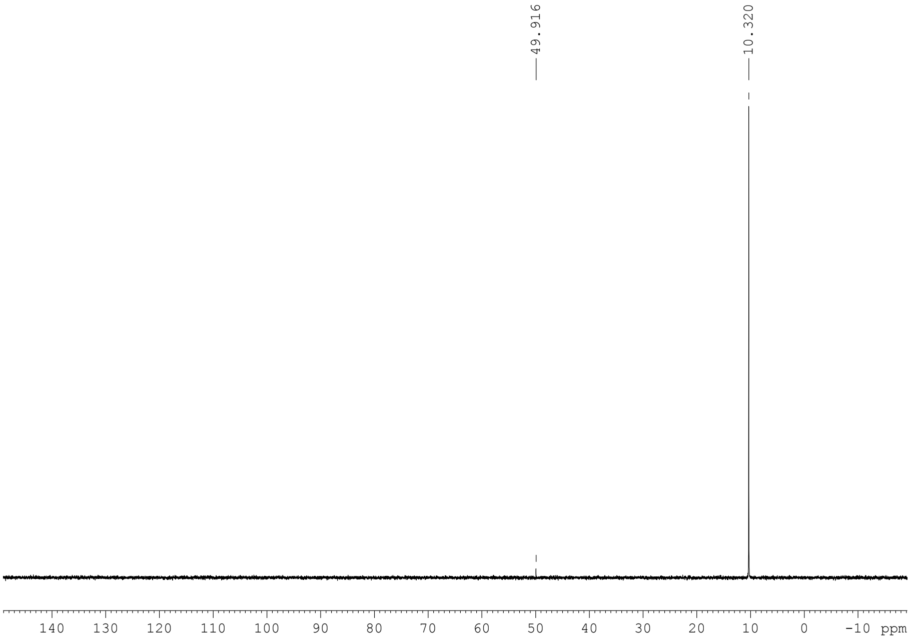

No exchange observed with complex **1**. Peak at 49.9 ppm might represent a transient species exchanging phosphine under such conditions.

5.7 ^{31}P-{^{1}H} NMR (C_7D_8) of complex 1 with 5 equiv PCy_3, 128 scans, 353 K

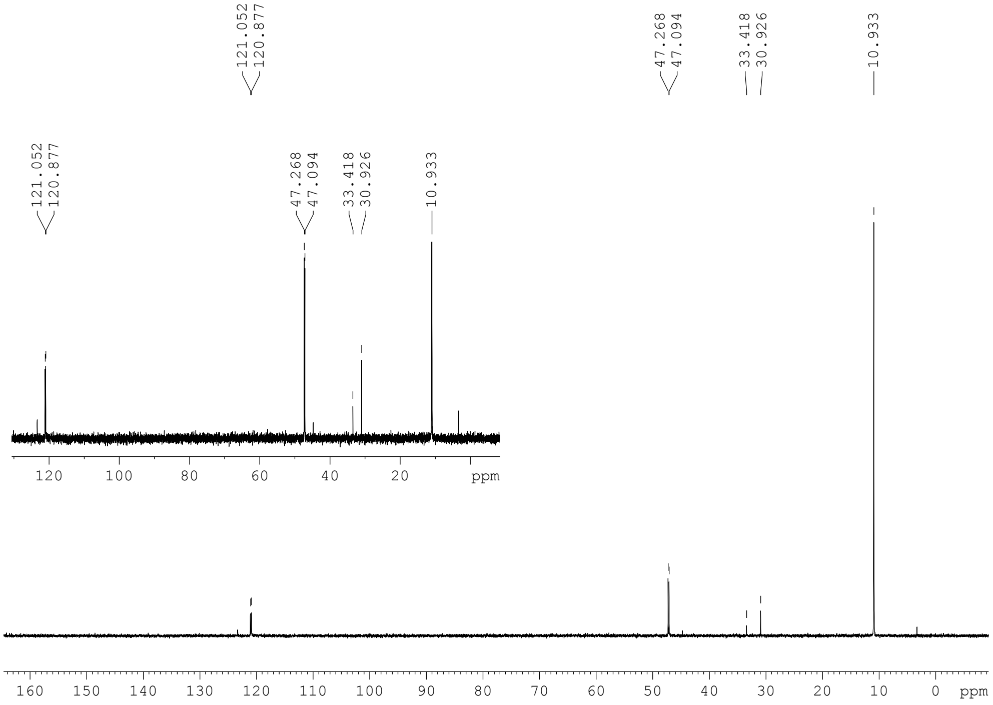

Reference spectrum of the NMR sample at 353 K. Some decomposition observed at 33.4 ppm.

5.8 ^{31}P EXSY (C_7D_8) of complex 1 with 5 equiv PCy_3, 128 scans, D8 = 0.6 s, 353 K

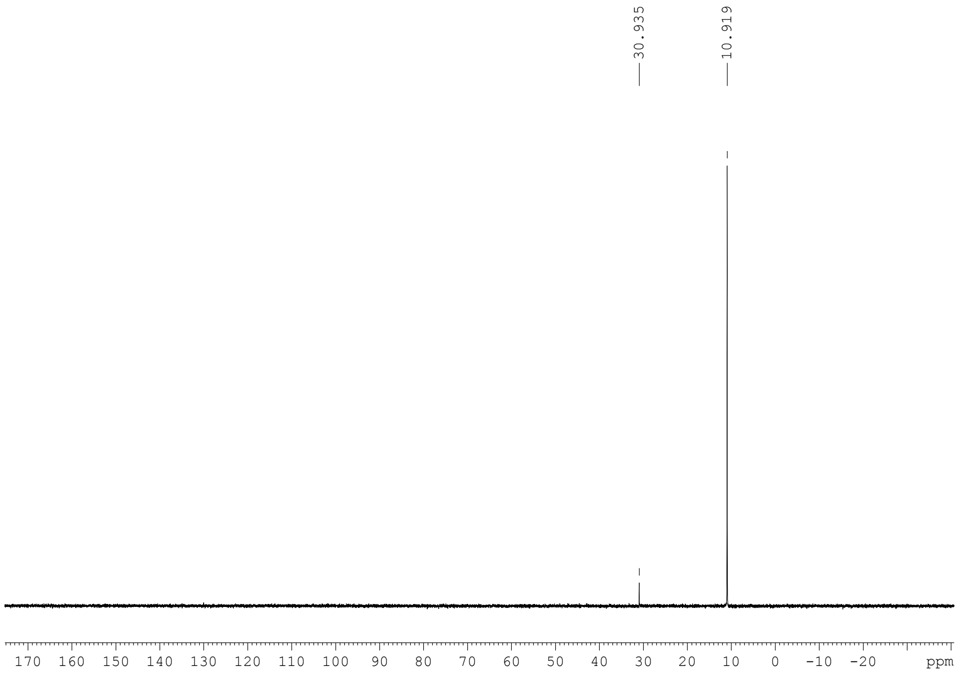

No exchange observed with complex **1**. Peak at 30.9 ppm might represent a transient species exchanging phosphine under such conditions, different from the previous one at 323 K.

5.9 ^{31}P EXSY (C_7D_8) of complex 1 with 5 equiv PCy_3, 128 scans, D8 = 1.2 s, 353 K

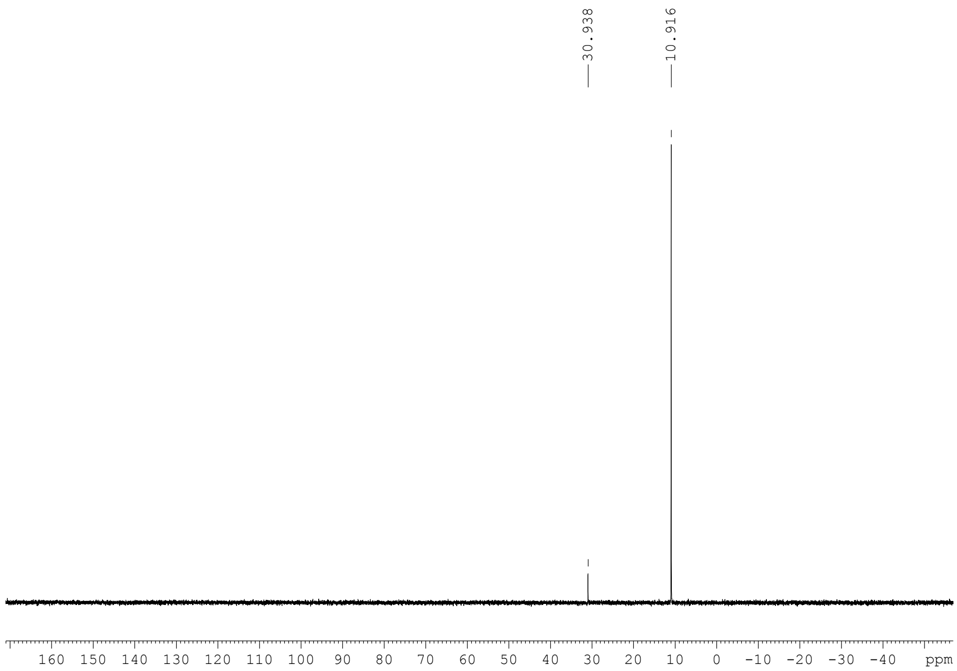

No exchange observed with complex **1**. Peak at 30.9 ppm might represent a transient species exchanging phosphine under such conditions, different from the previous one at 323 K.

5.10 ^{31}P EXSY (C_7D_8) of complex 1 with 5 equiv PCy_3, 512 scans, D8 = 0.6 s, 353 K

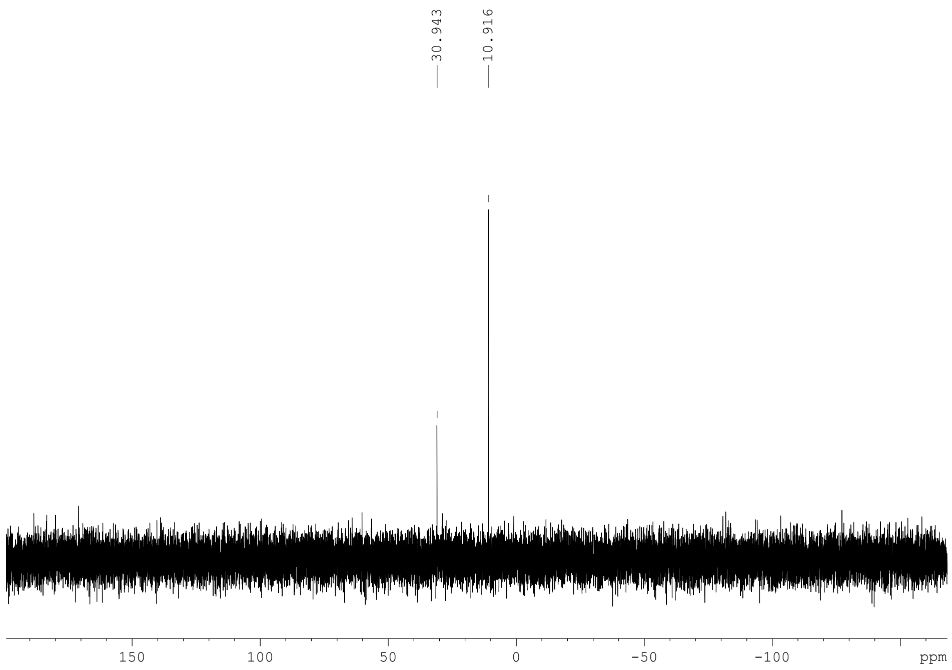

No exchange observed with complex **1**. Peak at 30.9 ppm might represent a transient species exchanging phosphine under such conditions, different from the previous one at 323 K.

5.11 ^{31}P EXSY (C_7D_8) of complex 1 with 5 equiv PCy_3, 512 scans, D8 = 1.2 s, 353 K

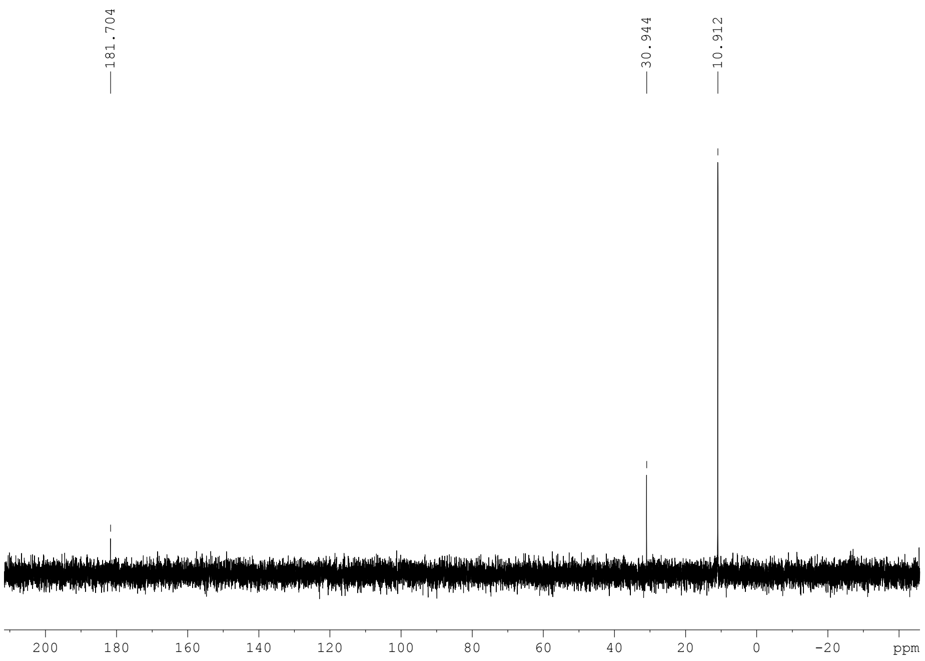

No exchange observed with complex **1**. Peaks at 30.9 and 181.7 ppm might represent transient species exchanging phosphine under such conditions, different from the previous ones at 323 K.

5.12 ^{31}P-{^{1}H} NMR (C_7D_8) of complex 1 with 5 equiv PCy_3, 128 scans, 293 K

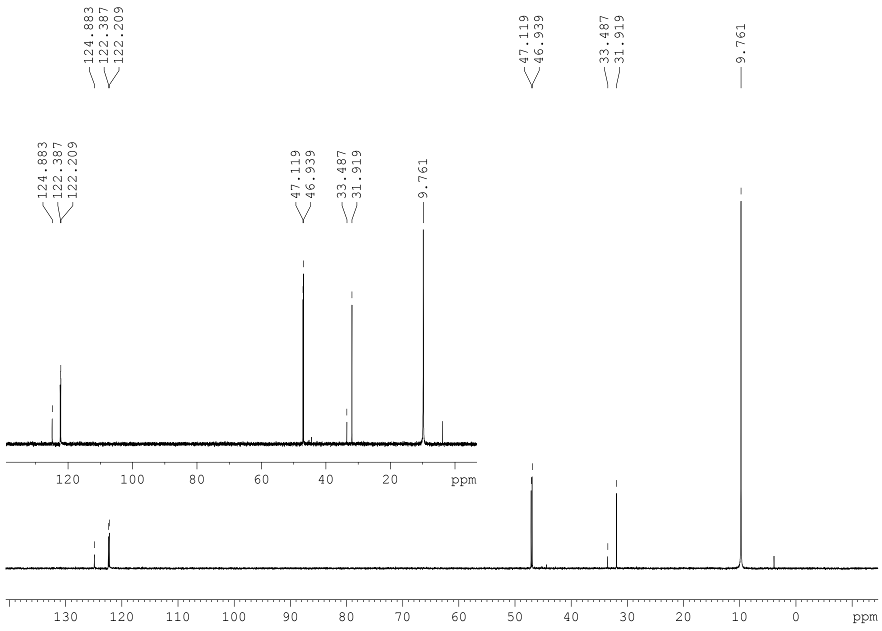

Final spectrum of the NMR sample back at 293 K. Some decomposition observed at 33.4 and 124.9 ppm.

6 HRMS analysis of product 3

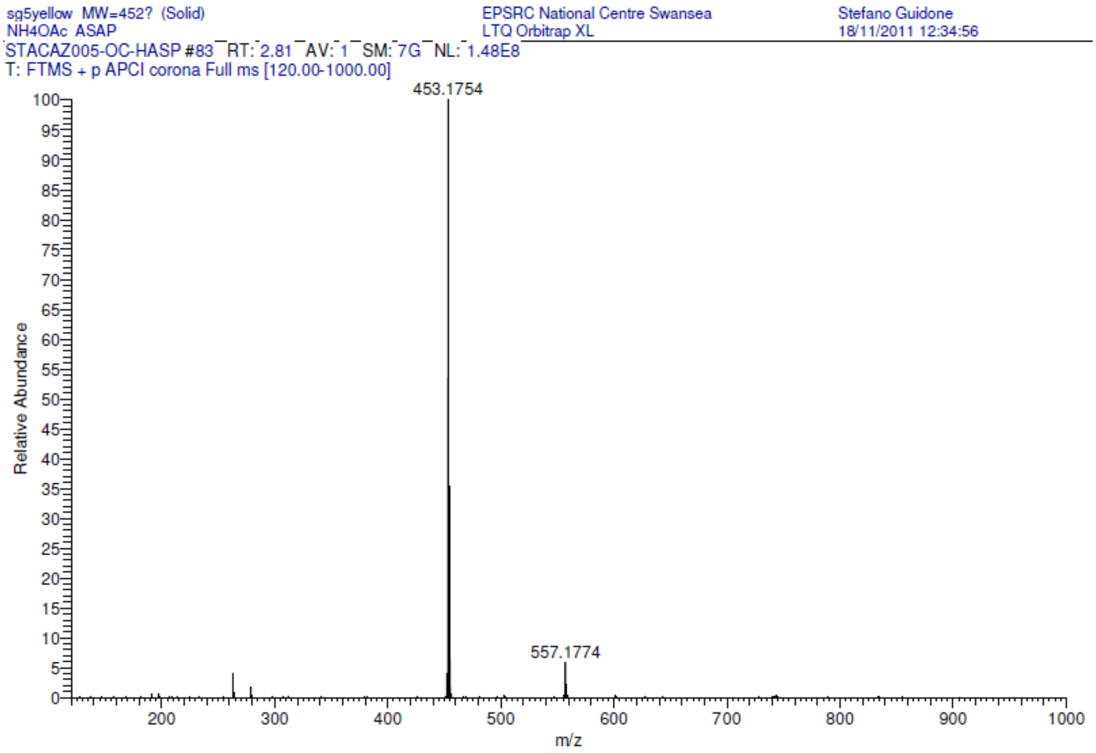

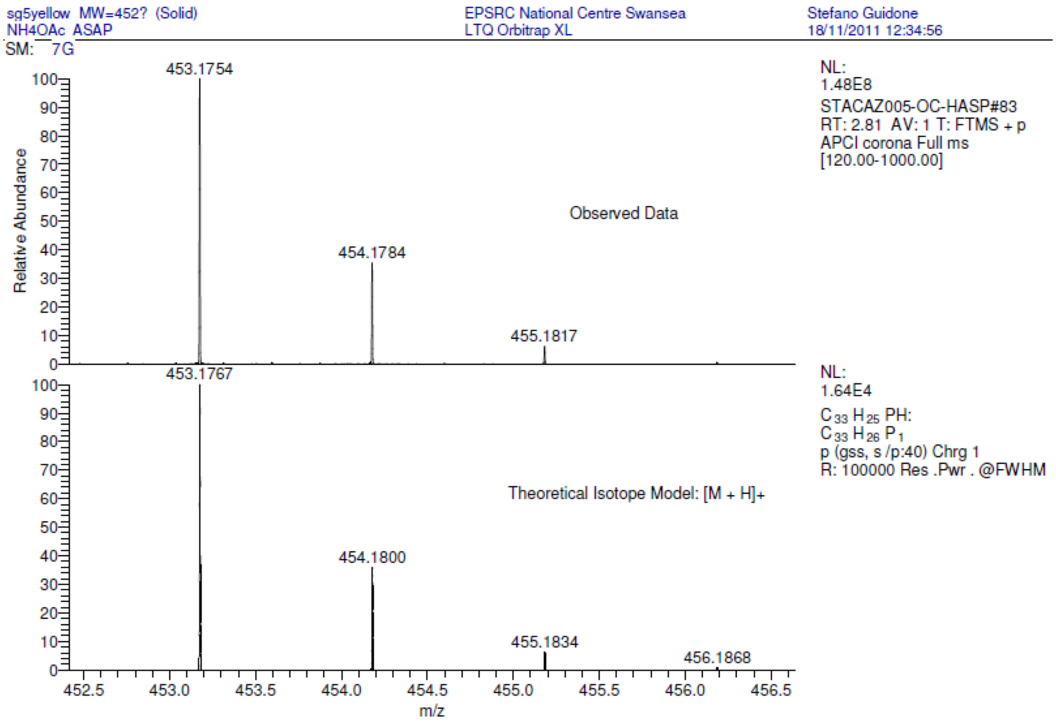

7 Crystal data and structure refinement

	CCDC/889638 **1**	CCDC/889639 **2**	CCDC/889640 **3**
Identification number	sgcc1	sgcc2	sgcc6
Empirical formula	C45 H71 Cl2 O3 P2 Ru	C33 H52 Cl2 O6 P2 Ru	C33.25 H25.50 Cl0.50 P
Formula weight	893.93	778.66	473.73
Temperature (K)	125(2)	93(2)	93(2)
Wavelength (Å)	0.71073	0.71073	0.71073
Crystal system	Orthorhombic	Orthorhombic	Orthorhombic
Space group	Pbca	Pbca	P2(1)2(1)2
Unit cell dimensions *a,b,c* (Å); α,β,γ (°)	13.703(2), 16.085(3), 41.003(7); 90, 90, 90	13.067(2), 15.910(2), 37.178(6); 90, 90, 90	18.848(4), 25.980(5), 10.209(2); 90, 90, 90
Volume ($Å^3$)	9038(3)	7729(2)	4999.1(18)
Z	8	8	8
Density (calcd) (Mg/m^3)	1.314	1.338	1.259
Absorption coeff. (mm^{-1})	0.573	0.665	0.184
F(000)	3784	3248	1988
Crystal size (mm^3)	0.18 x 0.14 x 0.03	0.20 x 0.20 x 0.02	0.05 x 0.05 x 0.05
Theta range for data collection (°)	0.99 to 25.45	2.30 to 25.38	2.14 to 25.38
Index ranges	-16<=h<=16, -19<=k<=17, -43<=l<=49	-12<=h<=15, -18<=k<=17, -44<=l<=41	-20<=h<=22, -31<=k<=31, -12<=l<=12
Reflections collected	67079	35597	50745
Independent reflections	8344 [R(int) = 0.1184]	6784 [R(int) = 0.0687]	9203 [R(int) = 0.1646]
Completeness to theta = 25.00°	99.7 %	95.9 %	99.9 %
Absorption correction	Semi-empirical from equivalents	Multiscan	Multiscan
Max. and min. transmission	1.000 and 0.548	1.000 and 0.791	1.000 and 0.558
Refinement method	Full-matrix least-squares on F^2	Full-matrix least-squares on F^2	Full-matrix least-squares on F^2
Data / restraints / parameters	8344 / 0 / 485	6784 / 0 / 397	9203 / 0 / 630
Goodness-of-fit on F^2	1.270	1.071	0.944
Final R indices [I>2sigma(I)]	R1 = 0.0730, wR2 = 0.1980	R1 = 0.0433, wR2 = 0.0891	R1 = 0.0796, wR2 = 0.2041
R indices (all data)	R1 = 0.0992, wR2 = 0.2295	R1 = 0.0642, wR2 = 0.0979	R1 = 0.1053, wR2 = 0.2199
Largest diff. peak and hole ($eÅ^{-3}$)	1.119 and -1.678	0.528 and -0.547	0.197 and -0.243

8 References

1. Bauer, C. J.; Freeman, R.; Frenkiel, T.; Keeler, J.; Shaka A. J. *J. Magn. Reson.* **1984**, *58*, 442–457.
